# Bioactivity of Serratiochelin A, a Siderophore Isolated from a Co-Culture of *Serratia* sp. and *Shewanella* sp.

**DOI:** 10.3390/microorganisms8071042

**Published:** 2020-07-14

**Authors:** Yannik Schneider, Marte Jenssen, Johan Isaksson, Kine Østnes Hansen, Jeanette Hammer Andersen, Espen H. Hansen

**Affiliations:** 1 Marbio, Faculty for Fisheries, Biosciences and Economy, UiT—The Arctic University of Norway, Breivika, N-9037 Tromsø, Norway; kine.o.hanssen@uit.no (K.Ø.H.); jeanette.h.andersen@uit.no (J.H.A.); espen.hansen@uit.no (E.H.H.); 2 Department of Chemistry, Faculty of Natural Sciences, UiT—The Arctic University of Norway, Breivika, N-9037 Tromsø, Norway; johan.isaksson@uit.no

**Keywords:** Serratiochelin A, Serratiochelin C, *Serratia* sp., siderophore, iron, anticancer, natural products, microbial biotechnology, degradation, antibacterial, *S. aureus*

## Abstract

Siderophores are compounds with high affinity for ferric iron. Bacteria produce these compounds to acquire iron in iron-limiting conditions. Iron is one of the most abundant metals on earth, and its presence is necessary for many vital life processes. Bacteria from the genus *Serratia* contribute to the iron respiration in their environments, and previously several siderophores have been isolated from this genus. As part of our ongoing search for medicinally relevant compounds produced by marine microbes, a co-culture of a *Shewanella* sp. isolate and a *Serratia* sp. isolate, grown in iron-limited conditions, was investigated, and the rare siderophore serratiochelin A (**1**) was isolated with high yields. Compound **1** has previously been isolated exclusively from *Serratia* sp., and to our knowledge, there is no bioactivity data available for this siderophore to date. During the isolation process, we observed the degradation product serratiochelin C (**2**) after exposure to formic acid. Both **1** and **2** were verified by 1-D and 2-D NMR and high-resolution MS/MS. Here, we present the isolation of **1** from an iron-depleted co-culture of *Shewanella* sp. and *Serratia* sp., its proposed mechanism of degradation into **2**, and the chemical and biological characterization of both compounds. The effects of **1** and **2** on eukaryotic and prokaryotic cells were evaluated, as well as their effect on biofilm formation by *Staphylococcus epidermidis*. While **2** did not show bioactivity in the given assays, **1** inhibited the growth of the eukaryotic cells and *Staphylococcus aureus*.

## 1. Introduction

Iron is the fourth most abundant metal in the Earth’s crust and is an absolute requirement for life [1]. Iron is an essential nutrient vital for several biological processes, such as respiration, gene regulation, and DNA biosynthesis [1,2]. Despite its abundance, iron is a growth-limiting factor for organisms in many environments [1]. To tackle this, microorganisms produce a vast range of iron-chelating compounds, called siderophores. Siderophores are compounds of low molecular weight (<1000 Da) that have high affinity and selectivity for ferric iron (iron(III)) [1], with the function of mediating iron uptake by microbial cells [3]. Siderophore production is commonly regulated by the iron concentration in the surroundings [4]. The siderophores are accumulated by membrane-bound iron receptors and brought inside the cell by active transport. Subsequently, the iron is normally reduced from iron(III) to iron(II). Since the affinity towards iron(II) is much lower than to iron(III), the iron is released from the iron-siderophore complex and can be utilized by the microorganism [4]. One of the major functional groups of siderophores is catecholate. Many siderophores of the catecholate type contain building blocks consisting of dihydroxybenzoic acid coupled to an amino acid [3]. The first catecholate-type siderophore, a glycine conjugate of 2,3-dihydroxybenzoic acid, was identified in 1958. The compound was produced by *Bacillus subtilis* under iron-poor conditions [5].

The genus *Serratia* is part of the family *Enterobacteriaceae*, whose type species is *Serratia marcescens* [6,7]. Species of the genus *Serratia* have been detected in diverse habitats, such as soil, humans, invertebrates, and water. For *Serratia plymuthica*, water appears to be the principal habitat [6]. Bacteria from this genus are also problematic in health care, as *Serratia marcescens* is an opportunistic pathogen causing infections in immunocompromised patients. One of the pathogenicity factors of the bacterium is its production of potent siderophores. Several different siderophores are produced by bacteria of this genus [8], one example being the serratiochelins produced by *Serratia* sp. V4 [9,10].

**Figure 1:**
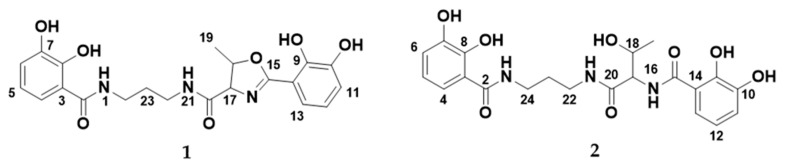
The structures of serratiochelin A (**1**) and C (**2**).

The serratiochelins are catecholate siderophores produced by *Serratia* sp. [9,10]. In a paper from 2012 by Seyedsayamdost and co-authors, a new siderophore biosynthetic pathway was proposed for the production of the serratiochelins [10]. The new pathway consisted of genes originating and recombined from two known siderophore biosynthetic clusters: The clusters for enterobactin (*Escherichia coli*) and vibriobactin (*Vibrio cholera*). The study mentions three different serratiochelins, serratiochelin A (**1**), B, and C (**2**); the structures of **1** and **2** can be seen in Figure 1. In the study from 2012, only two of the three compounds, **1** and serratiochelin B, were found in the untreated culture extracts, while **2** is a hydrolysis product of **1**, which was produced in the presence of formic acid. Sayedsayamdost et al. indicated that **1** and serratiochelin B were the native compounds produced by the bacterium [10].

Siderophores are of pharmaceutical interest. They can be used in their native form to treat iron overload diseases, like sickle cell disease. Desferal^®^ (Deferoxamine) is a siderophore-based drug used to treat iron poisoning and thalassemia major, a disease that leads to iron overload, which can lead to severe organ damage [11,12]. Siderophores can furthermore be used to facilitate active uptake of antibiotics by bacteria, and by the production of siderophore-antibiotic drug conjugates (SADCs). For some antibiotics, this strategy can reduce the minimal inhibitory concentration (MIC) by 100-fold, compared to an unbound antibiotic that enters the bacterial cell by passive diffusion [13]. The sideromycins are one example of SADCs. Albomycin, which belongs to this group, enters via the ferrichrome transporter, and has broad-spectrum antibiotic activity and is active against different Gram-negative bacteria [4,14]. The main problem with the use of SADCs is that most pathogenic bacteria have different routes for iron uptake, which could lead to higher frequency in resistance [4].

Due to the important role of *Shewanella* sp. and *Serratia* sp. in the environmental iron cycle, we were intrigued by observing a compound in high yields in an iron-limited co-culture of the two bacteria, which was not found in cultures supplemented with iron nor in axenic cultures of the bacteria. Here, we report the isolation of **1** from a co-culture of *Shewanella* sp. and *Serratia* sp. The degradation of **1** into **2** in the presence of acid was confirmed. To our knowledge, there is no published data regarding the bioactivity of these compounds. In this study, **1** and **2** were tested against a panel of bacterial and human cells, and for their ability to inhibit biofilm formation of the biofilm-producing bacterium *S. epidermidis*.

## 2. Materials and Methods

### 2.1. Bacterial Isolates

Compound **1** was isolated from bacterial cultures started from a non-axenic glycerol stock. The bacterial glycerol stock originally contained a *Leifsonia* sp. isolate. The stock was found to be contaminated with both *Shewanella* sp. and *Serratia* sp. after several steps of cultivation and production of new glycerol stock solutions. The non-axenic glycerol stock was inoculated onto three different agar plates, in order to gather information of the different isolates present. Originally, the *Leifsonia* sp. isolate was provided as an axenic culture by The Norwegian Marine Biobank (Marbank, Tromsø, Norway) (Reference number: M10B719). The bacterium was isolated from the intestine/stomach of an Atlantic hagfish (*Myxine glutinosa*) collected by benthic trawl in Hadselfjorden (Norwegian Sea, 16th of April, 2010). The bacterium was grown in liquid FMAP medium (15 g Difco Marine Broth (Becton Dickinson and Company, Franklin Lakes, NJ, USA), 5 g peptone from casein, enzymatic digest (Sigma, St. Louis, MS, USA), 700 mL ddH_2_O, and 300 mL filtrated sea water) until sufficient turbidity, and cryo-conserved at −80 °C with 30% glycerol (Sigma). Filtration of sea water was done through a Millidisk^®^ 40 Cartridge with a Durapore^®^ 0.22-µm filter membrane (Millipore, Burlington, MA, USA).

### 2.2. PCR and Identification of the Strains

The glycerol stock was plated onto three different types of agar: FMAP agar (FMAP medium with 15 g/L agar), DVR1 agar (6.7 g malt extract (Sigma), 11.1 g peptone from casein, enzymatic digest (Sigma), 6.7 g yeast extract (Sigma), 0.5 L filtered sea water, 0.5 L ddH_2_O), and potato glucose agar (Sigma). The plates with bacteria were incubated at 10 °C until sufficient growth, and transferred to 4 °C for temporary storage. This plating experiment resulted in the discovery of three different bacterial isolates, based on bacterial morphology and sequencing of the 16S rRNA gene. Clear colonies were picked from the plates, and inoculated into 100 µL of autoclaved ddH_2_O. The samples were stored at −20 °C until PCR amplification. The characterization of the bacterial strains was done with sequencing of the 16S rRNA gene through colony PCR and Sanger sequencing. The primer set used for amplification of the gene was the 27F primer (forward primer; 5′ - AGAGTTTGATCMTGGCTCAG) and the 1429R primer (reverse primer; 5′- TACCTTGTTACGACTT), both from Sigma. Prior to the amplification PCR, the bacterial samples were vortexed and diluted 1:100 and 1:1000 in UltraPure Water (BioChrom GmbH, Berlin, Germany). For PCR, 1 µL of the diluted bacterial sample was combined in a 25-µL PCR reaction, together with 12.5 µL DreamTaq Green PCR Master Mix (2×) (Thermo Scientific, Vilnius, Lithuania), 10.5 µL ultrapure water, and 0.5 µL of the forward and reverse primers (10 µM) mentioned above. The amplification was done using a Mastercycler ep gradient S (Eppendorf AG, Hamburg, Germany) with the following program: 95 °C initial denaturation for 3 min, followed by 35 cycles of 95 °C for 30 s, 47 °C for 30 s, and 72 °C for 1 min. Final extension was 72 °C for 10 min. The success and purity of the PCR reaction was analyzed on a 1.0% agarose gel (Ultrapure™ Agarose, Invitrogen, Paisley, UK) with Gel-Red^®^ Nucleic Acid Gel Stain (Biotium, Fremont, CA, USA), and the results were documented using a Syngene Bioimaging system (Syngene, Cambridge, UK). Successfully amplified samples were purified by the A’SAP PCR clean up kit (ArcticZymes, Tromsø, Norway). The purified PCR product was used for sequencing PCR, using 1 µL PCR product, 2 µL BigDye™ 3.1 (Applied Biosystems, Foster City, CA, USA), 2 µL 5× sequencing buffer (Applied Biosystems, Foster City, CA, USA), 4 µL of UltraPure water, and 1 µL of primer (1 µM of 27F primer or 1429R primer). The program for the sequencing PCR was as follows: 96 °C initial denaturation for 1 min, followed by 30 cycles of 96 °C for 10 s, 47 °C for 5 s, and 60 °C for 2 min. The PCR product was sequenced at the University Hospital of North Norway (Tromsø, Norway).

The forward and reverse sequences obtained were assembled using the Geneious Prime^®^ 2020.0.5 software (https://www.geneious.com). The sequences were assembled by using the built-in Geneious assembler. Prior to assembly, the sequences were trimmed using a 0.05 error probability limit. Sequence homology comparison was conducted using the built-in Basic Local Alignment Search Tool (BLAST) [15] in Geneious, excluding environmental samples, metagenomes, and uncultured microorganisms, for phylogenetic identification of the strains.

To identify which strain was responsible for the production of **1**, the three bacterial strains were isolated on separate agar plates and inoculated in small cultures of DVR1 medium (for media contents, see below). The bacteria were pelleted by centrifugation, and the supernatant was diluted 1:1 in methanol and ran on the UHPLC-HR-MS for identification of the compound.

### 2.3. Fermentation and Extraction of Bacterial Cultures

For extraction of compounds, the bacteria were cultivated in 1000-mL flasks containing 300 mL DVR1 medium (6.7 g malt extract (Sigma), 11.1 g peptone from casein, enzymatic digest (Sigma), 6.7 g yeast extract (Sigma), 0.5 L filtered sea water, and 0.5 L ddH_2_O) cultures for 16 days, at 10 °C and 130 rpm. A total of 12 flasks were inoculated, giving 3.6 L of culture. The medium was autoclaved for 30 min at 120 °C prior to inoculation. Cultures were started by loop inoculation from the non-axenic glycerol stock solution.

Extraction of metabolites from the liquid media was done with Diaion^®^ HP-20 resin (Supelco, Bellefonte, PA, USA). The resin was activated by incubation in methanol for 30 min, followed by washing with ddH_2_O for 15 min, and added to the cultures (40 g/L). The cultures were incubated with resin for 3 days prior to compound extraction. For extraction, the resin beads were separated from the liquid by vacuum filtration through a cheesecloth mesh (Dansk Hjemmeproduktion, Ejstrupholm, Denmark), the resin was washed with ddH_2_O, and finally extracted two times with methanol. The extract was vacuum filtered through a Whatman No. 3 filter paper (Whatman plc, Maidstone, UK), and dried under reduced pressure at 40 °C.

### 2.4. Fractionation by FLASH Chromatography

Due to the degradation of **1** in the presence of acid, the culture extract was fractionated for bioactivity testing and structure verification, using FLASH chromatography (Biotage SP4^TM^ system, Uppsala, SE), removing the use of acid in the purification process. The extract (3667.9 mg) was re-dissolved in 90% methanol, before adding Diaion^®^ HP20-SS resin (Supelco) in a ratio of 1:1.5 (resin:dry extract, *w*/*w*) and drying under reduced pressure at 40 °C. Due to the high amount of the extract, it was fractionated in two rounds. FLASH columns were prepared with 6.5 g activated Diaion^®^ HP-20SS resin per column. The dried extract was applied to the column, and ran with a water: methanol gradient from 5–100% methanol over 36 min at a flow rate of 12 mL/min. This resulted in 15 fractions per run. The fractions eluting at 100% methanol were analyzed on the UHPLC-HR-MS, and the purest fraction (fraction no 13, >95% pure based on UV/Vis) was used and dried under reduced pressure at 40 °C. The fraction yielded 50.9 mg and was used for the bioactivity testing.

### 2.5. UHPLC-HR-MS and Dereplication

UHPLC-HR-MS data for dereplication and to analyze the various experiments was recorded using an Acquity I-class UPLC (Waters, Milford, MA, USA) coupled to a PDA detector and a Vion IMS QToF (Waters). The chromatographic separation was performed using an Acquity C-18 UPLC column (1.7 µm, 2.1 mm × 100 mm) (Waters). Mobile phases consisted of acetonitrile (HiPerSolv, VWR, Radnor, PA, USA) for mobile phase B and ddH_2_O produced by the in-house Milli-Q^®^ system (Millipore, Burlington, MA, USA) as mobile phase A, both containing 1% formic acid (*v/v*) (33015, Sigma). The gradient was run from 10% to 90% B in 12 min at a flow rate of 0.45 mL/min. Samples were run in ESI+ and ESI- ionization mode. The data was processed and analyzed using UNIFI 1.9.4 (Waters). Exact masses and isotopic distributions were calculated using ChemCalc (https://www.chemcalc.org).

### 2.6. Purification by Preparative HPLC

Initially, the purification of **1** and **2** was done by preparative HPLC-MS using a 600 HPLC pump, a 3100 mass spectrometer, a 2996 photo diode array detector, and a 2767 sample manager (Waters). For infusion of the eluents into the ESI-quadrupole-MS, a 515 HPLC pump (Waters) and a flow splitter were used and 80% methanol in ddH_2_O (*v/v*) acidified with 0.2% formic acid (Sigma) as make-up solution at a flow rate of 0.7 mL/min. The columns used for isolation were a Sunfire RP-18 preparative column (10 µm, 10 mm × 250 mm) and XSelect CSH preparative fluoro-phenyl column (5 µm, 10 mm × 250mm), both columns were purchased from Waters. The mobile phases for the gradients were A (ddH_2_O with 0.1% (*v/v*) formic acid) and B (acetonitrile with 0.1% (*v/v*) formic acid), flow rate was set to 6 mL/min. Acetonitrile (Prepsolv^®^, Merck, Darmstad, Germany) and formic acid (33015, Sigma) were purchased in appropriate quality, ddH_2_O was produced with the in-house Milli-Q^®^ system. The collected fractions were reduced to dryness at 40 °C in vacuo and freeze drying using an 8L laboratory freeze dryer (Labconco, Fort Scott, KS, USA).

### 2.7. NMR analysis

NMR spectra were acquired in DMSO-*d*_6_ on a Bruker Avance III HD spectrometer (Bruker, Billerica, MA, USA) operating at 600 MHz for protons, equipped with an inverse TCI cryo probe enhanced for ^1^H, ^13^C, and ^2^H. All NMR spectra were acquired at 298 K, in 3-mm solvent-matched Shigemi tubes using standard pulse programs for proton, carbon, HSQC, HMBC, COSY, and ROESY with gradient selection and adiabatic versions where applicable. ^1^H/^13^C chemical shifts were referenced to the residual solvent peak (DMSO-*d*_6_: δH = 2.50, δC = 39.51).

### 2.8. Cultivation Study

Due to the hypothesis that the compound had iron-chelating properties for the bacteria, a cultivation study with and without the addition of iron to the medium was conducted. To investigate if the production was temperature specific, the bacteria were also grown at two different temperatures. The bacteria were grown in DVR1 medium and DVR2 medium (DVR1 with added 5.5 mL FeSO_4_ 7 H_2_O (8 g/L stock, ≙ 28.8 mM Fe)), at room temperature and at 10 °C with 130 rpm shaking. Samples were taken from the cultures, under sterile conditions, after 7, 14, and 21 days, for chemical analysis by UHPLC-HR-MS. From the cultures, 5 mL of sample were taken and centrifuged to pellet the bacteria, 1 mL of the supernatant was transferred to a new tube and centrifuged again, before sterile filtration using an Acrodisc syringe filter 0.2 µm, supor membrane (Pall Corp., East Hills, NY, USA) The filtered sample was mixed 1:1 with methanol prior to injecting on the UHPLC-HR-MS for investigation.

### 2.9. Marfey’s Amino Acid Analysis

A small quantity of **1** was dissolved in 1 mL of 6N HCL and incubated for 6 h at 110 °C using 1.5-mL reaction tubes and a thermoblock. After cooling down to room temperature, the reaction was reduced to dryness by vacuum centrifugation at 40 °C. The dry sample after hydrolysis was re-dissolved in 100 µL of H_2_O. The derivatization was carried out by mixing the re-dissolved hydrolystate with 180 µL FDAA in acetone (Marfey’s reagent, Sigma), N_α_-(2,4-Dinitro-5-fluorophenyl)-l-alaninamide), and 20 µL 1N NaHCO_3_. The reaction was incubated at 40 °C using a thermoblock. After incubation, the reaction was acidified with 30 µL of 1N HCl and diluted with 2.5 mL of methanol. Then, 0.1 mg of l-threonine and d-threonine dissolved in 100 µL water were used to prepare standards of the amino acids using the same derivatization procedure as described for the sample hydrolysate. The standards and sample diluted in methanol were analyzed using UHPLC-MS/MS as described above.

### 2.10. Iron Chelation Experiment

For testing the capability of **1** and **2** to chelate iron, a chelation assay was performed. The molecule was dissolved in water (0.2 mg/mL) and 75 µL of the molecule were mixed with 25 µL of 10 mg/mL FeCl_3_ × 6 H_2_O. The preparation was done in HPLC vials, the reaction was thoroughly mixed by vortexing, centrifuged, and subsequently analyzed by UHPLC-MS/MS.

### 2.11. Hydrolyzation with Formic Acid

For testing the liability for hydrolyzation, a 1-mg sample of **1** was dissolved in 1 mL 10% (*v/v*) DMSO *aq*. and incubated for 24 h at room temperature with formic acid concentrations of 0% (control), 0.1%, 1.0%, 5.0%, and 10% (*v/v*). The reaction product was analyzed by UHPLC-MS/MS.

### 2.12. Production of Serratiochelin C

For testing the bioactivity of **2** in comparison to **1**, a sample of non-degraded **1** was hydrolyzed by adding 10% (*v*/*v*) formic acid and incubation over 24 h at room temperature. The formic acid was removed by vacuum centrifugation at 40 °C and subsequent freeze drying using a laboratory freeze dryer (Labconco).

### 2.13. Bioactivity Testing

#### 2.13.1. Growth Inhibition Assay

To determine antimicrobial activity, a bacterial growth inhibition assay was executed. Compounds **1** and **2** were tested against *Staphylococcus aureus* (ATCC 25923), *Escherichia coli* (ATCC 259233), *Enterococcus faecialis* (ATCC 29122), *Pseudomonas aeruginosa* (ATCC 27853), *Streptococcus agalactiae* (ATCC 12386), and Methicillin-resistant *Staphylococcus aureus* (MRSA) (ATCC 33591), all strains from LGC Standards (Teddington, London, UK). *S. aureus*, MRSA, *E. coli*, and *P. aeruginosa* were grown in Muller Hinton broth (275730, Becton). *E. faecalis* and *S. agalactiae* were cultured in brain hearth infusion broth (53286, Sigma). Fresh bacterial colonies were transferred to the respective medium and incubated at 37 °C overnight. The bacterial cultures were diluted to a culture density representing the log phase and 50 µL/well were pipetted into a 96-well microtiter plate (734-2097, Nunclon™, Thermo Scientific, Waltham, MA, USA). The final cell density was 1500–15,000 colony forming units/well. The compound was diluted in 2% (*v/v*) DMSO (Dimethyl sulfoxide) in ddH_2_O, and the final assay concentration was 50% of the prepared sample, since 50 µL of sample in DMSO/water were added to 50 µL of bacterial culture. After adding the samples to the plates, they were incubated over night at 37 °C and the growth was determined by measuring the optical density at λ = 600 nm (OD_600_) with a 1420 Multilabel Counter VICTOR3™ (Perkin Elmer, Waltham, MA, USA). A water sample was used as the reference control, growth medium without bacteria as a negative control, and a dilution series of gentamycin (32 to 0.01 µg/mL, A2712, Merck) as the positive control and visually inspected for bacterial growth. The positive control was used as a system suitability test and the results of the antimicrobial assay were only considered valid when the positive control was passed. The final concentration of DMSO in the assays was ≤2% (*v/v*), known to have no effect in the tested bacteria. The data was processed using GraphPad Prism 8 (GraphPad, San Diego, CA, USA).

#### 2.13.2. Cell Proliferation Assay

The inhibitory effect **1** and **2** was tested using an MTS in vitro cell proliferation assay against two cell lines: The human melanoma cell line A2058 (ATCC, CLR-1147™), and for general cytotoxicity assessment, the non-malignant MRC5 lung fibroblast cells (ATCC CCL-171™) were employed. The cells were cultured and assayed in Roswell Park Memorial Institute medium (RPMI-16040, FG1383, Merck) containing 10% (*v/v*) fetal bovine serum (FBS, 50115, Biochrom, Holliston, MA, USA). The cell concentration was 4000 cells/well for the lung fibroblast cells and 2000 cells/well for the cancer cells. After seeding, the cells were incubated for 24 h at 37 °C and 5% CO_2_. The medium was then replaced with fresh RPMI-1640 medium supplemented with 10% (*v/v*) FBS and gentamycin (10 µg/mL, A2712, Merck). After adding 10 µL of sample diluted in 2% (*v/v*) DMSO in ddH_2_O, the cells were incubated for 72 h at 37 °C and 5% CO_2_. For assaying the viability of the cells, 10 µL of CellTiter 96^®^ AQueous One Solution Reagent (G3581, Promega, Madison, WI, USA) containing tetrazolium [3-(4,5-dimethylthiazol-2-yl)-5-(3-carboxymethoxyphenyl)-2-(4-sulfophenyl)-2*H*-tetrazolium, inner salt] and phenazine ethosulfate was added to each well and incubated for one hour. The tests were executed with three technical replicates. The plates were read using a DTX 880 plate reader (Beckman Coulter, CA, USA) by measuring the absorbance at λ = 485 nm. The cell viability was calculated using the media control. As a negative control, RPMI-1640 with 10% (*v/v*) FBS and 10% (*v/v*) DMSO (Sigma) was used as a positive control. The data was processed and visualized using GraphPad Prism 8.

#### 2.13.3. Biofilm Inhibition Assay

For testing the inhibition of biofilm formation, the biofilm-producing *Staphylococcus epidermidis* (ATCC 35984) was grown in Tryptic Soy Broth (TSB, 105459, Merck, Kenilworth, NJ, USA) overnight at 37 °C. The overnight culture was diluted in fresh medium with 1% glucose (D9434, Sigma) before being transferred to a 96-well microtiter plate; 50 µL/well were incubated overnight with 50 µL of the test compound dissolved in 2% (*v/v*) DMSO aq. added in duplicates. The bacterial culture was removed from the plate and the plate was washed with tap water. The biofilm was fixed at 65 °C for 1 h before 70 µL of 0.1% crystal violet (115940, Millipore) were added to the wells for 10 min of incubation. Excess crystal violet solution was then removed and the plate dried for 1 h at 65 °C. Seventy microliters of 70% ethanol were then added to each well and the plate incubated on a shaker for 5–10 min. Biofilm formation inhibition were assessed by the presence of violet color and was measured at 600-nm absorbance using a 1420 Multilabel Counter VICTOR3™. Fifty microliters of a non-biofilm-forming *Staphylococcus haemolyticus* (clinical isolate 8-7A, University Hospital of North Norway Tromsø, Norway) mixed in 50 µL of autoclaved Milli-Q water was used as a control; 50 µL of *S. epidermidis* mixed in 50 µL of autoclaved Milli-Q water was used as the control for biofilm formation; and 50 µL of TSB with 50 µL of autoclaved Milli-Q water was used as a medium blank control.

## 3. Results

Compound **1** was isolated from a co-culture of *Serratia* sp. and *Shewanella* sp. when cultivated in an iron-limited medium. The bacteria were also cultivated in iron-supplemented media, where **1** was not detected. Compound **1** was only produced in co-cultures started directly from the glycerol stock by loop inoculation, and not found in any axenic cultures. The cultures were extracted, and the extracts were fractionated using FLASH chromatography to isolate serratiochelin A (**1**), a siderophore previously isolated exclusively from a *Serratia* sp., also when grown under iron-limited conditions [10]. During preparative HPLC-MS isolation, it was observed that the compound was degraded, and the degradation product was found to be serratiochelin C (**2**), which corresponds to previous observations [10]. A study of the iron binding of the compounds and a degradation study with formic acid was conducted. The structures of the compounds were verified by 1-D and 2-D NMR and MS experiments, and Marfey’s analysis was used to find the configuration of the threonine moiety of the molecule. Compound **1** and **2** were tested for their antibacterial activities, their abilities to inhibit the formation of biofilm, and their toxicity towards human cells. This is the first study on the bioactivity of **1** since its original discovery in 1994 [9].

### 3.1. Identification of Co-Culture and Serratiochelin A Production Strain

When streaking out the glycerol stock onto three different agar plates, three morphologically different bacterial colonies were observed (Figure S14). The 16S rRNA gene of these bacteria was amplified and sequenced by Sanger sequencing, showing that the stock solution contained *Leifsonia* sp. (original isolate in stock), *Shewanella* sp., and *Serratia* sp. The 16S rRNA sequences for the three isolates can be found in the Supplementary Material (Texts S15–S17). *Shewanella* sp. and *Serratia* sp. are assumed to be of marine origin, as strains of the same genera have been cultivated at the same time as the *Leifsonia* sp. isolate, and the 16S rRNA sequences are similar to two strains of the Marbank strain collection (*Shewanella* sp. M10B851 and *Serratia* sp. M10B861, Marbank ID). In order to investigate if all bacteria were able to co-exist in the liquid culture started from the glycerol stock, a 450-mL culture of DVR1 was inoculated with the glycerol stock (identically as was done with the culture from which **1** was isolated) and the culture was streaked out on agar after 3 and 10 days of cultivation. After three days of cultivation, the colony forming units (CFUs) of both *Shewanella* sp. and *Serratia* sp. were observed on the plates (Figure S14), proven by morphological identification and sequencing of the 16S rRNA gene. After 10 days of culturing, no CFUs of *Shewanella* sp. were observed from the culture, and the experiment detected exclusively CFUs of *Serratia* sp. No *Leifsonia* colonies were observed from the liquid cultures, not after 3 nor 10 days of cultivation. This indicates that the *Serratia* sp. isolate outgrow the other two isolates in the cultivation done in this study. After re-streaking the three bacterial isolates present in the glycerol stock to obtain pure cultures, the different isolates were cultivated separately in 50-mL cultures in DVR1 to identify the actual producer of **1**. Compound **1** was only produced in co-cultures started directly from the glycerol stock, and not by any of the cultures started from axenic colonies from agar plates.

### 3.2. Dereplication and Isolation

Serratiochelin A (**1**) was obtained as a brown powder. The bacterial extracts and fractions were analyzed using UHPLC-IMS-MS and **1** was detected at *m/z* 430.1594 ([M+H]^+^) in ESI+ eluting at 4.45 min. The calculated elemental composition was C_21_H_23_N_3_O_7_ (Calc. *m/z* 430.1614 [M+H]^+^), corresponding to 12 degrees of unsaturation. The elemental composition gave several hits for natural products in available databases, including serratiochelin A (**1**). As **1** had been previously isolated from *Serratia* sp., we saw it as a clear possibility that we had a positive identification of the compound. However, to confirm this, isolation and structure elucidation was necessary. After the first round of isolation using preparative HPLC, we detected two species of the product, one at RT= 4.45 min (**1**) and another at RT = 2.07 min (**2**), both having the same *m/z* and elemental composition in ESI+. We later confirmed that the *m/z* of **2** in ESI+ was not the *m/z* of the molecular ion due to neutral water loss in the ion source. The masses of **1** and **2** are thus not equivalent, which was later confirmed by ESI- ionization, which confirmed the mass of **2** to be equal to that of **1**+H_2_O.

**Figure 2:**
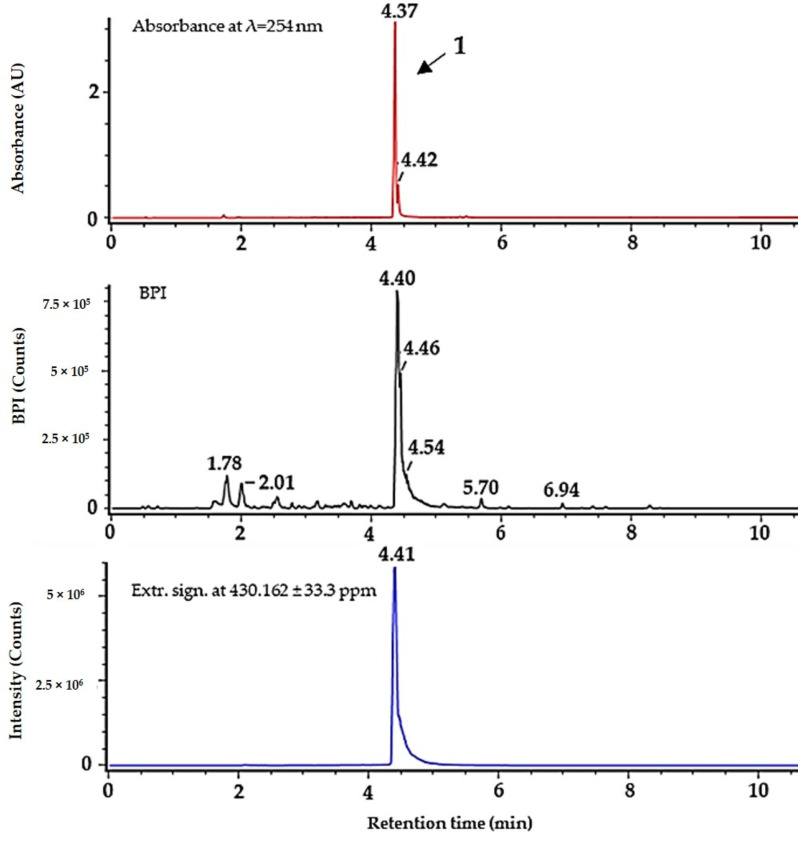
Purity of serratiochelin A (**1**) after isolation using FLASH chromatography, analyzed by UHPLC-MS. Top (in red) absorbance at 254 nm, middle (black) BPI chromatogram, bottom (blue) extracted signal for *m/z* = 430.162 (±33.3 ppm). ΔRT for UV/Vis detector is ~−0.05 min.

It was not possible to obtain **1** as a pure compound after the purification, as it was always accompanied by **2**, indicating a possible degradation of **1**. Compound **2,** on the other hand, was obtained as a pure compound after using preparative HPLC for isolation. To distinguish between the two molecules, the collision cross section (CCS) and drift time of the compounds were compared, and the samples were also investigated in ESI- (see Table 2 for the respective values, the high- and low-energy MS spectra, as well as UV/Vis spectra for **1** and **2** that are given in Figures S11 and S12). For isolating **1,** FLASH chromatography was used, since there was no **2** detected using this protocol, where no acid was employed. The collected fractions were assayed individually using UHPLC-MS and the first fraction eluting at 100% methanol was found to be sufficiently pure for structure elucidation via NMR and further bioactivity testing (results of the purity assay are given in Figure 2), yielding 50.9 mg **1** from 3667.9 mg of extract. Compound **1** was not readily dissolved in water and methanol but it dissolved in DMSO. Solutions of **1** were prepared in 100% DMSO and further diluted in water. The same was done with **2,** which also dissolved in methanol.

Serratiochelin C (**2**) was obtained as a brown powder, after acid-catalyzed degradation of **1**. From the ESI-, it was possible to elucidate the elemental composition of **2**. Compound **2** was detected, with *m/z* 446.1568 ([M-H]^-^) in ESI- eluting at 2.07 min. The calculated elemental composition was C_21_H_25_N_3_O_8_ (Calc. *m/z* 446.1563 [M-H]^-^), corresponding to 11 degrees of unsaturation.

### 3.3. Structure Elucidation

**Figure 3:**
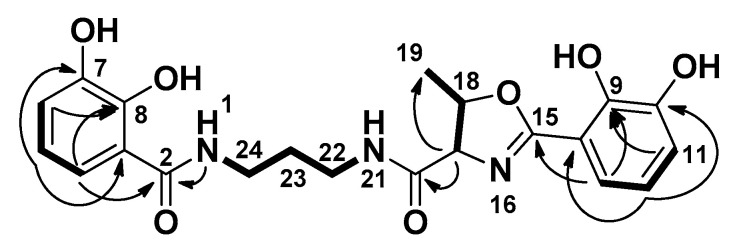
Key COSY (bold) and HMBC (arrow) correlations for serratiochelin A (**1**).

Close inspection of 1-D (^1^H and ^13^C, Table 1) and 2-D (HSQC, HMBC, COSEY, and ROESY) NMR data of **1** confirmed that we isolated the previously reported compound serratiochelin A (**1**). All NMR spectra can be seen in the Supplementary Material (Figures S1–S5). Key COSY and HMBC correlations used to assign the structure of **1** can be seen in Figure 3.

In preparations treated with formic acid, we detected a third molecule eluting at 2.60 min. According to its signal, fragments, and retention time, we concluded it was serratiochelin B [10]. Serratiochelin B was not isolated or verified by NMR. Serratiochelin B and **2** were not present within the crude extract or within fractions obtained by FLASH chromatography but were detected after treatment with acid. The conformation of threonine was found to be L by Marfey’s method, which is in compliance what has been published previously [10]. Results are given within the Supplementary Material (Figure S13).

### 3.4. Detection of Iron Chelation

Compounds **1** and **2** were mixed with aqueous FeCl_3_ solution to investigate if the compounds were able to chelate iron. Both **1** and **2** chelated iron, and the mass spectrometric data given in Table 2 indicate chelation of iron by the loss of three protons through coordination, as published previously [10]. The calculated exact mass for chelation of **1** was *m/z* 483.0729 ([M+Fe-2H]^+^) and for **2** and serratiochelin B *m/z* 501.0835 ([M+Fe-2H]^+^). In ESI-, the calculated *m/z* ratios were *m/z* 481.0572 ([M+Fe-4H]^+^) for **1** and *m/z* 499.0678 ([M+Fe-4H]^+^) for **2**.

### 3.5. Degradation Study with Formic Acid

**Figure 4:**
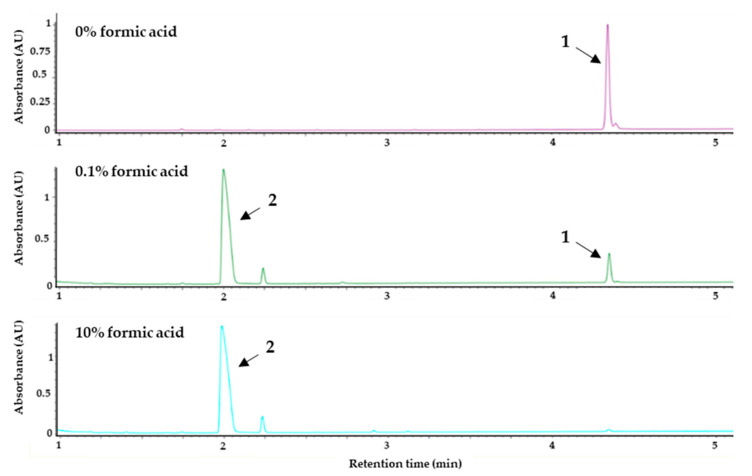
UV-max plot chromatogram. Degradation study showing the effect of formic acid on serratiochelin A (**1**). The purified sample of **1** was treated with different concentrations of formic acid (% (*v/v*)) for 24 h at room temperature and subsequently analyzed via UHPLC-PDA-MS. The chromatograms of the control (0% formic acid), 0.1% formic acid, and 10% formic acid are given above. The degradation of **1** (RT = 4.45 min) into serratiochelin C (**2**) (RT = 2.07 min) corresponds to the amount of formic acid used.

**Figure 5:**
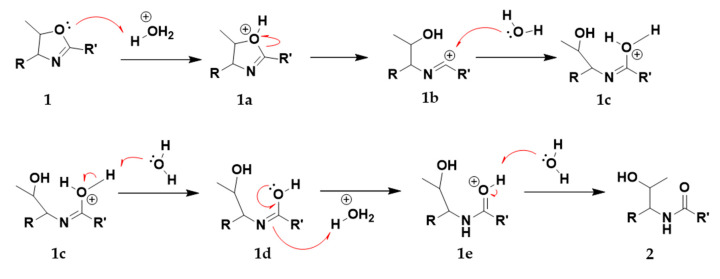
The proposed acid-catalyzed degradation reaction of the central methylated oxazoline ring of **1**, turning **1** into **2** via intermediates **1a** to **1e**.

The study confirmed that the degradation was triggered by formic acid. In order to obtain a pure sample of **1**, we used FLASH fraction no. 13, which predominantly contained **1** since during the extraction process and the FLASH chromatography, no formic acid or acidic solution is used that could induce degradation. This sample was used for the degradation study. Formic acid at concentrations of 0.1%, 1.0%, 5.0%, and 10% (*v/v*) were tested and compared to the control (no acid), as can be seen in Figure 4. It was found that the degradation correlates with the concentration of formic acid. The degradation takes place not only in the presence of formic acid. When incubated with 1% (*v/v*) hydrochloric acid or acetic acid, we observed degradation to approximately the same extent (data not shown). The acid-catalyzed degradation mechanism turning **1** into **2** via intermediates **1a**–**e** can be seen in Figure 5.

### 3.6. Cultivation Study

**Figure 6:**
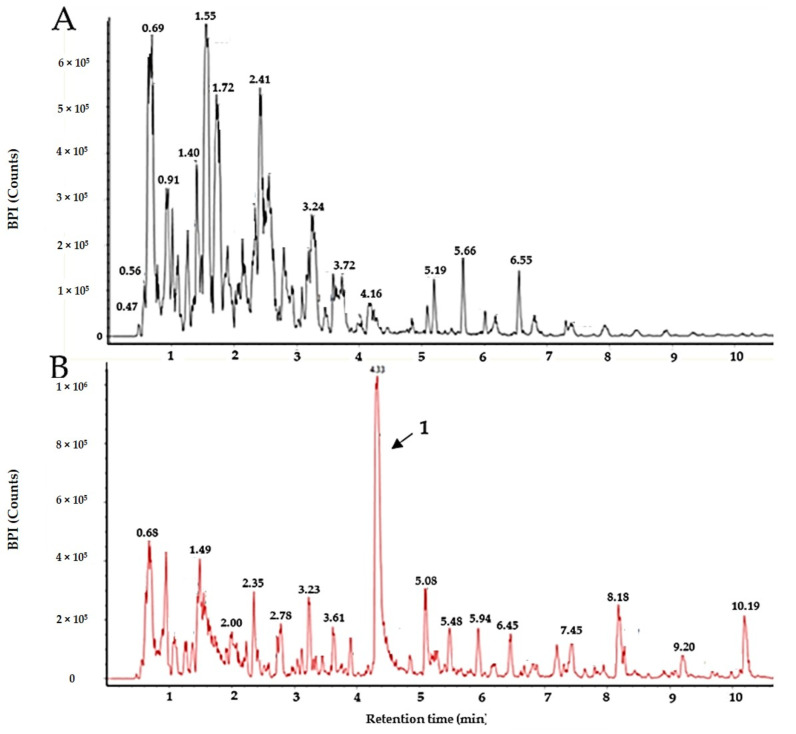
BPI chromatograms of the extracts of two co-cultures. (**A**) The extract of a 14-day culture (10 °C) supplemented with 160 µM Fe(III). (**B**) The extract of a 14-day culture (10 °C) grown in iron-deficient media. The peak of serratiochelin A (**1**) is indicated by the black arrow.

The cultivation study revealed that **1** was only produced in the iron-deficient co-cultures, as can be seen in Figure 6. Cultures grown in media supplemented with 160 µM FeSO_4_ did not produce **1** after 7, 14, and 21 days when grown at room temperature nor when grown at 10 °C (Figure 6). Within the iron-deficient cultures, **1** was detected after 7, 14, and 21 days cultivation at 10 °C as well as when cultivated at room temperature. Additionally, when extracting two cultures grown for 14 days at 10 °C using solid-phase extraction, there was no **1** present within the iron-supplemented media while it was a major component in the extract of the iron-deficient culture. Serratiochelin B and **2** were not detected in the cultures, nor in crude extracts after solid-phase extraction.

### 3.7. Bioassays

**Figure 7:**
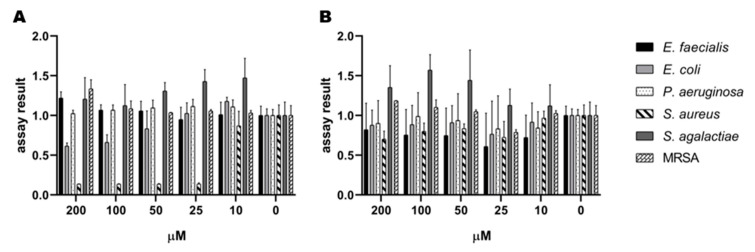
Initial screen of antibacterial activity of (**A**) serratiochelin A (**1**) and (**B**) serratiochelin C (**2**) on *E. faecialis*, *E. coli*, *P. aeruginosa*, *S. agalactiae*, and MRSA, normalized assay results. The experiment was executed twice with two technical replicates each.

**Figure 8:**
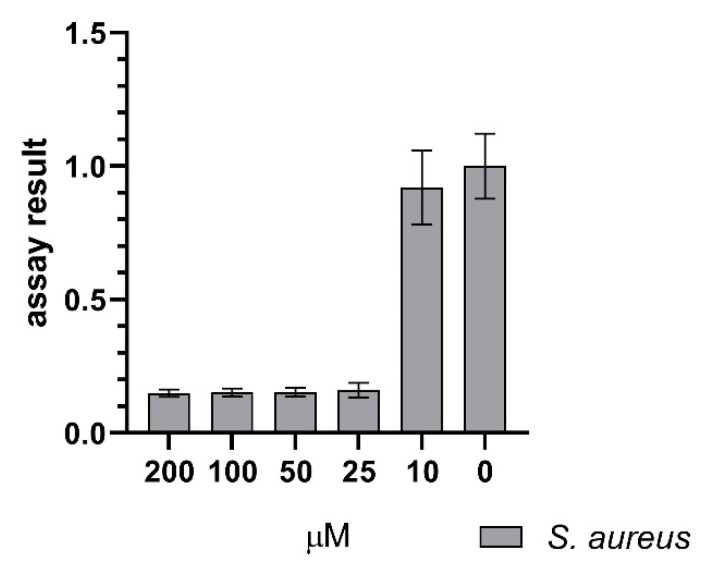
Effect of serratiochelin A (**1**) on *S. aureus* showing inhibition of growth down to 25 µM, normalized assay results. The assay was executed in four experiments with 3 × 2 and 1 × 3 technical replicates.

The growth-inhibiting properties of **1** and **2** were tested against several Gram-positive and Gram-negative strains. The antimicrobial assay detected an effect of **1** on *S. aureus*. Interestingly, there was no effect of **2** on *S. aureus* detected in the assay. There was no antimicrobial effect of **1** and **2** against *S. agalactiae*, *P. aeruginosa*, *E. coli*, *E. faecalis*, and MRSA observed. The results against all the test strains can be seen in Figure 7. The antimicrobial assay with *S. aureus* was repeated to verify the effect of **1**. Among the tested concentrations, 25 µM was the lowest concentration of **1**, which completely inhibited the growth of *S. aureus*, as displayed in Figure 8. Compound **1** and **2** were also tested for their ability to inhibit biofilm formation by *S.epidermidis* in concentrations up to 200 µM. Compound **1** showed some weak effects (assay result of ~ 40%, meaning 60% inhibition, normal cut-off used for further investigation is minimum 70% inhibition) at 200 µM. Compound **2** showed no visible effect up to 200 µM.

**Figure 9:**
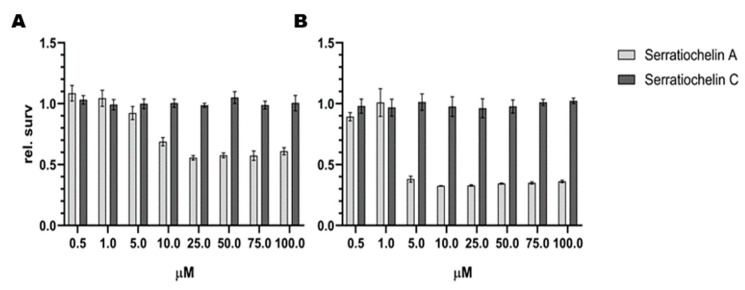
Antiproliferative effect of serratiochelin A (**1**) and C (**2**) on (**A**) A2058 (melanoma) and (**B**) MRC-5 (non-malignant lung fibroblasts) cell lines. The experiments were repeated twice with three technical replicates.

The effects of the compounds on eukaryotic cells was evaluated using the human melanoma cell line A2058 and the non-malign lung fibroblast cell line MRC5, see Figure 9. The effect of **2** on both cell lines is insufficient, while **1** reduces the cell proliferation of both MRC5 and A2058 cells. The effect of **1** is stronger against MRC5 cells than against A2058.

## 4. Discussion

In this study, a siderophore was isolated from a co-culture of a *Shewanella* sp. and *Serratia* sp. bacteria, both of which come from bacterial genera that are important for environmental iron metabolism. Bacteria from the genus *Shewanella* are known for their important role in iron metabolism, especially in aquatic environments. Previously, several siderophores have been isolated from bacteria of the genus *Serratia*, among these serratiochelin A (**1**).

*Shewanella* is a genus of Gram-negative rod-shaped γ-proteobacteria, within the order Alteromonadales, found mostly in aquatic habitats [16]. Bacteria from this genus have been isolated from several aquatic sources, both marine and freshwater [17,18,19,20]. The genus was established in 1985 [21], after a reconstruction of the *Vibrionaceae* family. *Shewanella* is part of the monogeneric family Shewanellaceae [16], which consists only of this one genera. The genus has high respiratory diversity, with the capability to respire approximately 20 different compounds, including toxic compounds and insoluble metals, one example being reducing Fe(III) chelate and Fe(III) oxide to produce soluble Fe(II) [22]. Bacteria from this genus are often involved in the iron metabolism in their environment, and several iron chelators (siderophores) have been isolated from this genus. Putrebactin is a cyclic dihydroxamate siderophore, produced and isolated from *S. putrefaciens* [23].

To investigate if the three bacterial isolates present in the glycerol stock co-exist in the liquid DVR1 cultures, the culture was streaked out on several agar plates after 3 and 10 days of incubation. The *Shewanella* colonies appeared first, followed by *Serratia* forming colonies on top of the *Shewanella* sp. colonies (Figure S14). After 10 days, there were only colony forming units of *Serratia* sp. present from the liquid co-culture, and the *Shewanella* could not be detected when streaked out on agar. Serratiochelins have previously only been isolated from the *Serratia* genus, and are considered to be rare siderophores [10]. As *Serratia* completely dominates the *Serratia*-*Shewanella* co-culture after 10 days, and based on data reported regarding previous isolation of **1 [9,10]**, it seems to be reasonable to hypothesize that *Serratia* is the true producer of **1** in this co-culture and that it is outcompeting *Shewanella* because of its specific iron acquisition. As **1** was not observed in axenic cultures of *Shewanella* or *Serratia*, we assume that the co-culturing is inducing the production of the compound, possibly due to the competition for iron in the culture.

Compound **1** was isolated from the co-culture after modifying the purification protocol. The degradation of **1** to **2** was triggered by formic acid used in the mobile phase during chromatographic isolation of the compounds. We confirmed that the degradation correlates with the concentration of formic acid as previously published [10]. In addition, the same acidic hydrolyzation of an oxazoline ring was also observed for the compound agrobactin after exposure to hydrochloric acid [24]. We also confirmed the chelation of iron in a hexadecanoate coordination indicated by the loss of three protons, observed in HR-MS experiments [10]. Compound **1** was only produced when no additional iron was added to the co-culture. In the presence of iron, **1** was not detected in the bacterial culture media. We did not detect serratiochelin B and **2** in the culture media, extract, or FLASH fractions (where no acid was used). Previously, it was reported that **1** and serratiochelin B are the initial biosynthetic products of *Serratia* [10]. For our isolate, the results strongly indicate that **1** is the only biosynthetic product, while serratiochelin B and **2** are degradation products of **1**. To obtain **1**, its liability for acid degradation is a significant disadvantage. The FLASH liquid chromatography represents a rather inefficient method for isolation since we were taking only the fraction with the highest purity. Thus, a considerable amount of compound eluted before and after together with other impurities, which diminished the yield of pure **1** significantly, and the purification protocol was not optimized regarding yields but for obtaining **1** without its degradation product. We assume that within the producer isolates’ natural environment, **1** is, however, most likely not degrading into **2** due to the rather alkaline pH of seawater [25].

The acid-free isolation enabled us to isolate **1** for bioactivity testing. Since there is no bioactivity data present for **1** and **2,** and the purpose of our investigation was to find new bioactive molecules, it was prioritized for isolation. The testing of both compounds revealed some interesting insights into their bioactivity. Compound **2** displayed no activity in the tested assays and at the tested concentrations, while **1** had antibacterial activity against *S. aureus* and toxic effects on both eukaryotic cell lines tested. Its antibacterial effect was specific towards *S. aureus*, while not having an effect on the other bacteria, including MRSA. Its cytotoxic effect was evaluated against the melanoma cell line (A2058), as we frequently observed that it is the most sensitive cancer cell line in our screening of extracts and compounds. The non-malignant lung fibroblasts (MRC5) was included as a general control of toxicity. The observed effect was stronger on lung fibroblasts than melanoma cells. Of interest to us was the observed difference in activity between **1** and **2** despite the fact that the two structures are closely related. It is questionable if the antiproliferative effect of **1** is caused by iron deprivation as observed for other siderophores [26] or by another effect. The same applies for the observed antibacterial effect on *S.aureus*, while the lack of effect on the other bacteria might indicate a specific target. Both molecules are capable of chelating iron, so either **1** has a higher affinity to iron than **2**, or it has another mode of action. The species-specific antibacterial effect indicates the latter. Gokarn and co-authors investigated the effect of iron chelation by exochelin-MS, mycobactin S, and deferoxiamine B on mammalian cancer cell lines and an antiproliferative effect was observed at concentrations between 0.1 to 1.0 mg/mL. Only HEPG2 cells have shown 23% cell survival at 20 µg/mL for mycobactin S. They observed a different sensitivity among the tested cell lines and siderophores [26]. Compound **1** had an effect at concentrations of <43 µg/mL (40% cell survival was detected at 2.15 µg/mL of **1** against MRC5). Therefore, testing of **1** against more cell lines and testing of **2** at higher concentrations would be an approach for further studies on the antiproliferative effects of **1** and **2**. Some siderophores are known to have additional functions, such as a virulence factor and modulation of the host of a pathogen [27]. Assuming another mode of action than iron chelation, the most relevant structural difference would be the 5-methyl-2-oxazoline heterocycle in **1**, which is hydrolyzed in **2**. Oxazole and oxazoline moieties are structural motives present in molecules with an antibacterial and antiproliferative effect [28,29]. They are ligands to a number of different protein targets and can be regarded as “privileged structures” [29,30]. Further bioactivity elucidation of the two serratiochelins and the mode of action studies of **1** will be the subject of further investigation.

## 5. Conclusions

We proved the production of **1** in high yields by a co-culture of *Serratia* sp. and *Shewanella* sp., while the compound was not observed in axenic cultures. We confirmed the iron chelation, as well as the degradation of **1** to **2**. We did not observe the production of any compound that could be related to serratiochelin B in the bacterial cultures nor in the extract, but we observed its generation in traces during acid-induced degradation, which gives rise to the assumption that serratiochelin B and **2** are both hydrolyzation products of **1** in this study.

While **1** showed antiproliferative activity on human cancer cells but also on non-malignant lung fibroblasts, and a specific antimicrobial effect on *S. aureus*, **2** did not show any bioactivity in the assays conducted in this study. Since **1** and **2** differ in the presence of a structural motif that can be seen as a privileged structure, we hypothesize that the hydrolyzation of the 5-methyl-2-oxazoline explains the difference in bioactivity. The liability for hydrolyzation, however, represents a strong disadvantage for developing this candidate further as a drug lead.

## Figures and Tables

**Table 1:** ^1^H- and ^13^C-NMR data for serratiochelin A (**1**) and C (**2**) in DMSO-*d*_6_.

NMR Data	Serratiochelin A (1)		Serratiochelin C (2)	
Position	δ_C_, Type	δ_H_ (*J* in Hz)	δ_C_, Type	δ_H_ (*J* in Hz)
1		8.30, t (5.9)		8.01, t (5.9)
2	169.6, C		169.73, C	
3	114.9, C		114.92, C	
4	117.1, CH	7.25, dd (8.1, 1.5)	117.04, CH	7.25, dd (8.2, 1.5)
5	117.8, CH	6.67, t (7.9)	117.84, CH	6.66, t (8.0)
6	118.7, CH	6.9, dd (7.8, 1.4)	118.65, CH	6.89, dd (7.8, 1.5)
7	146.3, C		146.27, C	
8	149.8, C		149.81, C	
9	148.3, C		146.12, C	
10	145.7, C		148.27, C	
11	119.4, CH	6.96, dd (7.8, 1.6)	118.18, CH	6.92, dd (7.7, 1.5)
12	118.7, CH	6.73, t (7.9)	117.77, CH	6.69, t (7.9)
13	117.9, CH	7.07, dd (7.9, 1.6)	118.92, CH	7.37, dd (8.1, 1.6)
14	110.3, C		116.77, C	
15	165.7, C		168.01, C	
16				8.66, s
17	73.7, CH	4.45, d (87.3)	59.18, CH	4.34, dd (8.0, 4.4)
18	78.8, CH	4.86, p (6.4)	66.38, CH	4.10, qd (6.1, 4.7)
19	20.7, CH_3_	1.45, d (6.3)	20.30, CH_3_	1.09, d (6.4)
20	169.8, C		169.99, CH	
21		8.81, s		8.78, t (5.3)
22	36.7, CH_2_	3.30, m	36.58, CH_2_	3.29, q (6.7)
23	28.9, CH_2_	1.72, p (7.0)	28.96, CH_2_	1.67, p (7.0)
24	36.6, CH_2_	3.18, m	36.41, CH_2_	3.20–3.08, m

The structure of serratiochelin C (**2**) was confirmed in a similar manner to that of **1**. All NMR spectra can be seen in the Supplementary Material (Figures S6–S10).

**Table 2:** IMS and MS data for the apo- and ferrylspecies of serratiochelin A (**1**), serratiochelin B, and serratiochelin C (**2**).

Compounds	Form	Ionization	RT ^*^ [min]	*m/z*	CCS ^**^ [A^2^]	Drift Time [ms]
Serratiochelin C (earliest eluting)	apo	ESI+	2.07	430.1610 ^***^	202.88	7.00
	apo	ESI-	2.05	446.1568 ^***^	198.35	6.94
	ferri	ESI+	2.09	501.0844	208.06	6.75
	ferri	ESI-	2.11	499.0680	203.54	7.12
Serratiochelin B (middle eluting)	apo	ESI+	2.64	448.1714	210.99	6.84
	apo	ESI-	2.60	446.1573	199.52	6.98
	ferri	ESI+	2.61	501.0822	211.91	6.89
	ferri	ESI-	2.62	499.0673	201.16	7.04
Serratiochelin A (late eluting)	apo	ESI+	4.45	430.1611	202.85	6.99
	apo	ESI-	4.37	428.1466	201.47	7.05
	ferri	ESI+	4.46	483.0723	206.88	6.70
	ferri	ESI-	4.39	481.5058	208.50	7.30

^*^ Retention time, ^**^ Collision cross section, ^***^ Loss of water of apo-serratiochelin C (**2**) in ESI+, not in ESI-, and not for the ferri-siderophores.

## Supplementary Materials

The following are available online at https://www.mdpi.com/2076-2607/8/7/1042/s1, Figure S1: ^1^H NMR (600 MHz, DMSO-*d*_6_) spectrum of **1**; Figure S2: ^13^C (151 MHz, DMSO-*d*_6_) spectrum of **1**; Figure S3: HSQC + HMBC (600 MHz, DMSO-*d*_6_) spectrum of serratiochelin A; Figure S4: COSY (600 MHz, DMSO-*d*_6_) spectrum of **1**; Figure S5: ROESY (600 MHz, DMSO-*d*_6_) spectrum of **1**; Figure S6: ^1^H NMR (600 MHz, DMSO-*d*_6_) spectrum of **2**; Figure S7: ^13^C (151 MHz, DMSO-*d*_6_) spectrum of **2**; Figure S8: HSQC + HMBC (600 MHz, DMSO-*d*_6_) spectrum of **2**; S9: HSQC + HMBC (600 MHz, DMSO-*d*_6_) spectrum of **2** (**2**), zoomed in crowded area; Figure S10: COSY (600 MHz, DMSO-*d*_6_) spectrum of **2**; Figure S11: Mass spectra of **1** and **2**; Figure S12: UV/Vis spectra of **1**and **2**; Figure S13: Chromatograms of Marfey’s analysis of **1**; Figure S14: Isolation of the bacteria and co-culture of *Serratia* sp. and *Shewanella* sp., Text S15: Consensus sequence of *Shewanella* sp.; Text S16: Consensus sequence of *Serratia* sp.; Text S17: Consensus sequence of *Leifsonia* sp.
