# Supplementary material for: Bioactivity of Serratiochelin A, a Siderophore Isolated from a Co-Culture of Serratia sp. and Shewanella sp
Source: Microorganisms. 2020 Jul 14;8(7):1042. doi: 10.3390/microorganisms8071042 (PMC7409175; doi:10.3390/microorganisms8071042)
Supplement: Supplementary file 1 [file microorganisms-08-01042-s001.docx]

Bioactivity of Serratiochelin A, a Siderophore Isolated from a Co-Culture of *Serratia* sp. and *Shewanella* sp.

Yannik Schneider ^1†*^, Marte Jenssen ^1†*^, Johan Isaksson ^2^, Kine Ø. Hansen ^1^, Jeanette Hammer Andersen ^1^ and Espen H. Hansen ^1^

^1^ Marbio, Faculty for Fisheries, Biosciences and Economy, UiT—The Arctic University of Norway, Breivika, N-9037 Tromsø, Norway; kine.o.hanssen@uit.no (K.Ø.H.); espen.hansen@uit.no (E.H.H.); jeanette.h.andersen@uit.no (J.H.A.)

^2^ Department of Chemistry, Faculty of Natural Sciences, UiT—The Arctic University of Norway, Breivika, N-9037 Tromsø, Norway; johan.isaksson@uit.no (J.I.)

* Correspondence: yannik.k.schneider@uit.no; Tel.: +47-77649267; marte.jenssen@uit.no; Tel.: +47-77649275

† Authors contributed equally to the work

Supplemental Information Table of Contents

**NMR Spectroscopic results**

**Serratiochelin A (1)**

**Figure S1** ^1^H NMR (600 MHz, DMSO-*d*_6_) spectrum of serratiochelin A (**1**)

**Figure S2** ^13^C (151 MHz, DMSO-*d*_6_) spectrum of serratiochelin A (**1**)

**Figure S3** HSQC + HMBC (600 MHz, DMSO-*d*_6_) spectrum of serratiochelin A (**1**)

**Figure S4** COSY (600 MHz, DMSO-*d*_6_) spectrum of serratiochelin A (**1**)

**Figure S5** ROESY (600 MHz, DMSO-*d*_6_) spectrum of serratiochelin A (**1**)

**Serratiochelin C (2)**

**Figure S6** ^1^H NMR (600 MHz, DMSO-*d*_6_) spectrum of serratiochelin C (**2**)

**Figure S7** ^13^C (151 MHz, DMSO-*d*_6_) spectrum of serratiochelin C (**2**)

**Figure S8** HSQC + HMBC (600 MHz, DMSO-*d*_6_) spectrum of serratiochelin C (**2**)

**Figure S9** HSQC + HMBC (600 MHz, DMSO-*d*_6_) spectrum of serratiochelin C (**2**), zoomed in crowded area

**Figure S10** COSY (600 MHz, DMSO-*d*_6_) spectrum of serratiochelin C (**2**)

**Results chemistry and mass spectrometry**

**Figure S11** Mass spectra of serratiochelin A (**1**) and serratiochelin C (**2**)

**Figure S12** UV/Vis spectra of serratiochelin A (**1**) and serratiochelin C (**2**)

**Figure S13** Chromatograms of Marfey’s analysis of serratiochelin A (**1**)

**Pictures of culture plates**

**Figure S14** Isolation of the bacteria and co-culture of *Serratia* sp. and *Shewanella* sp.

**Consensus sequences of the bacterial isolates**

**Text S15:** Consensus sequence of *Shewanella* sp.

**Text S16:** Consensus sequence of *Serratia* sp.

**Text S17:** Consensus sequence of *Leifsonia* sp


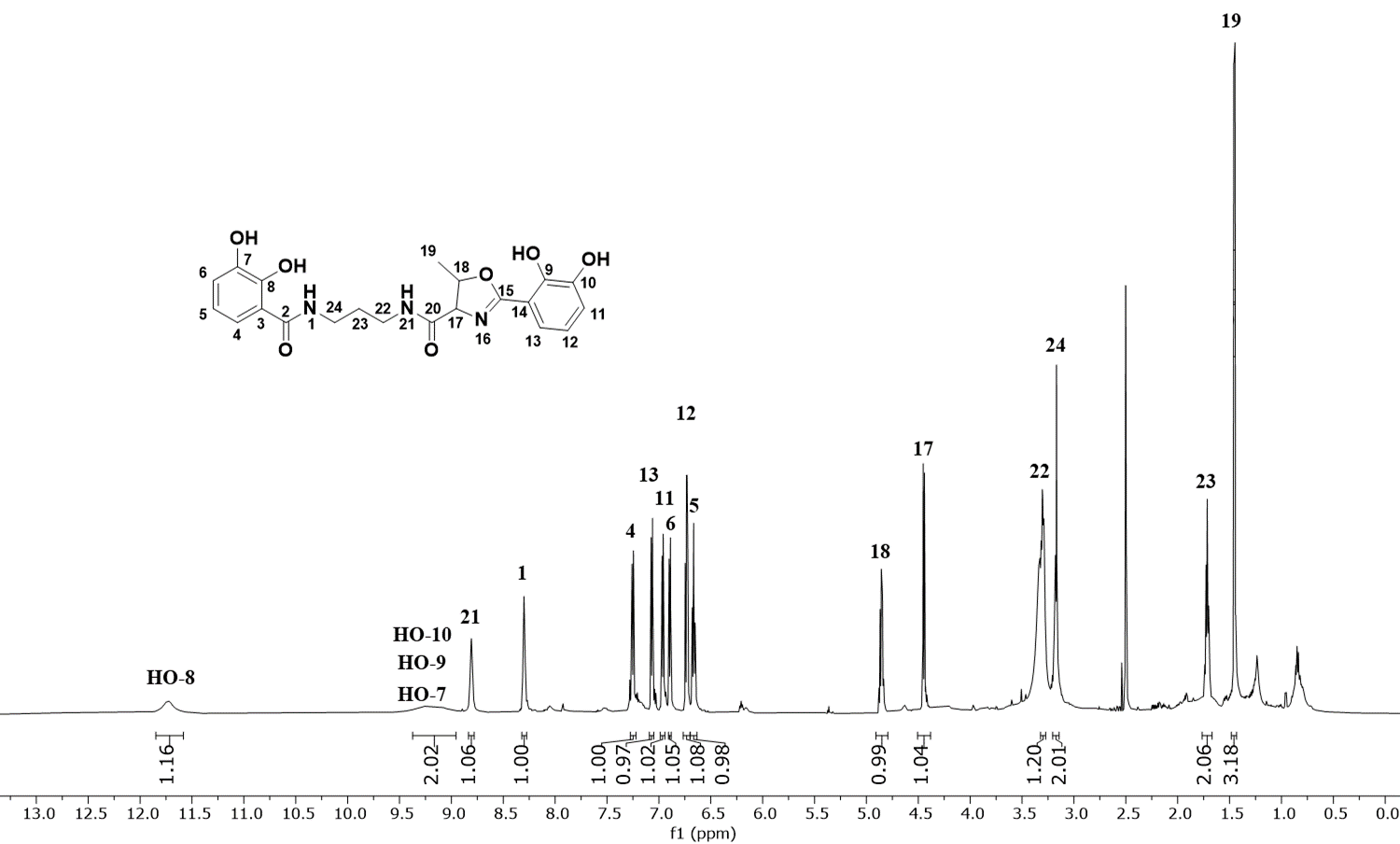


**S1.** ^1^H NMR (600 MHz, DMSO-*d*_6_) spectrum of serratiochelin A (**1**)


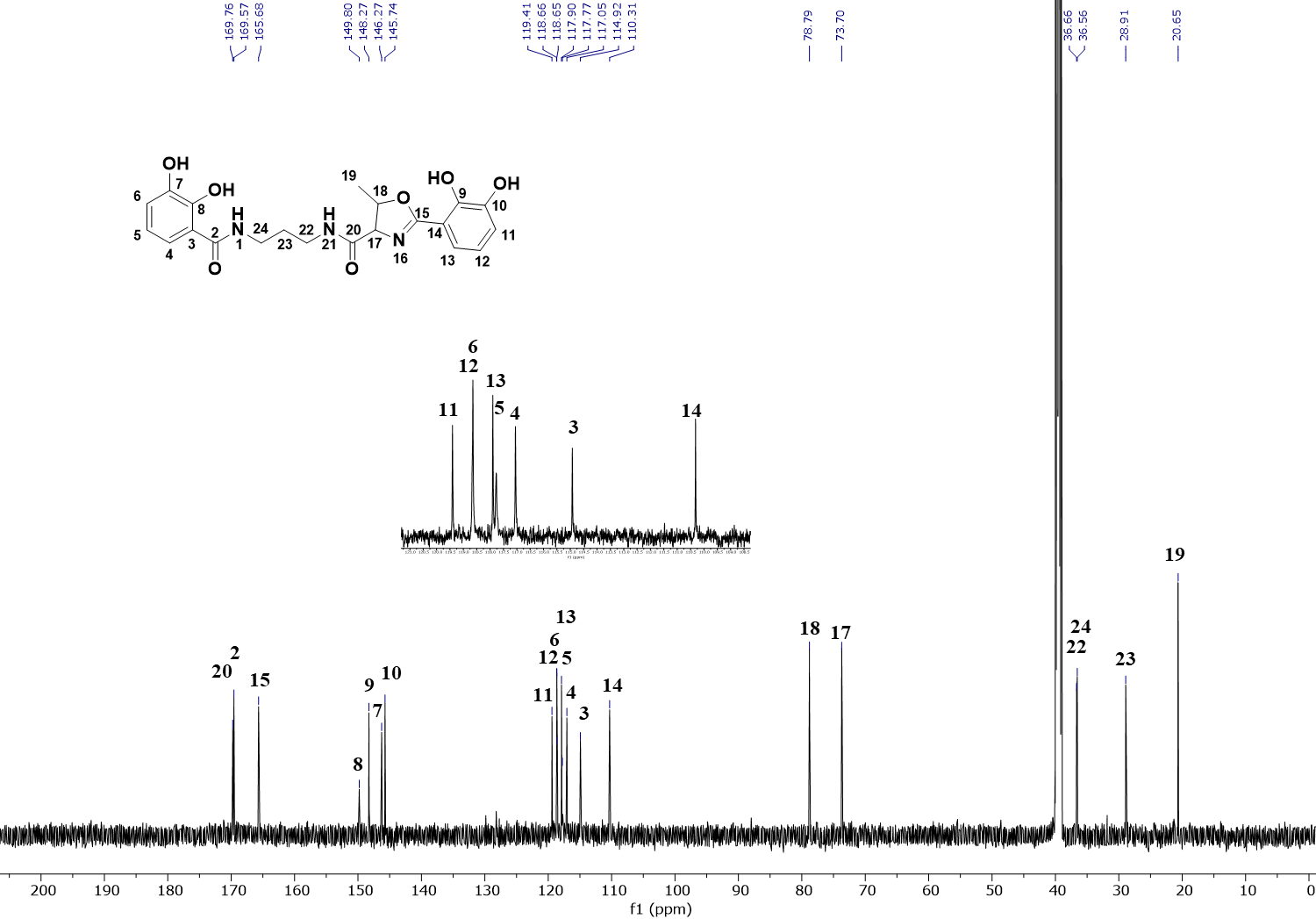


**S2.** ^13^C (151 MHz, DMSO-*d*_6_) spectrum of serratiochelin A (**1**)


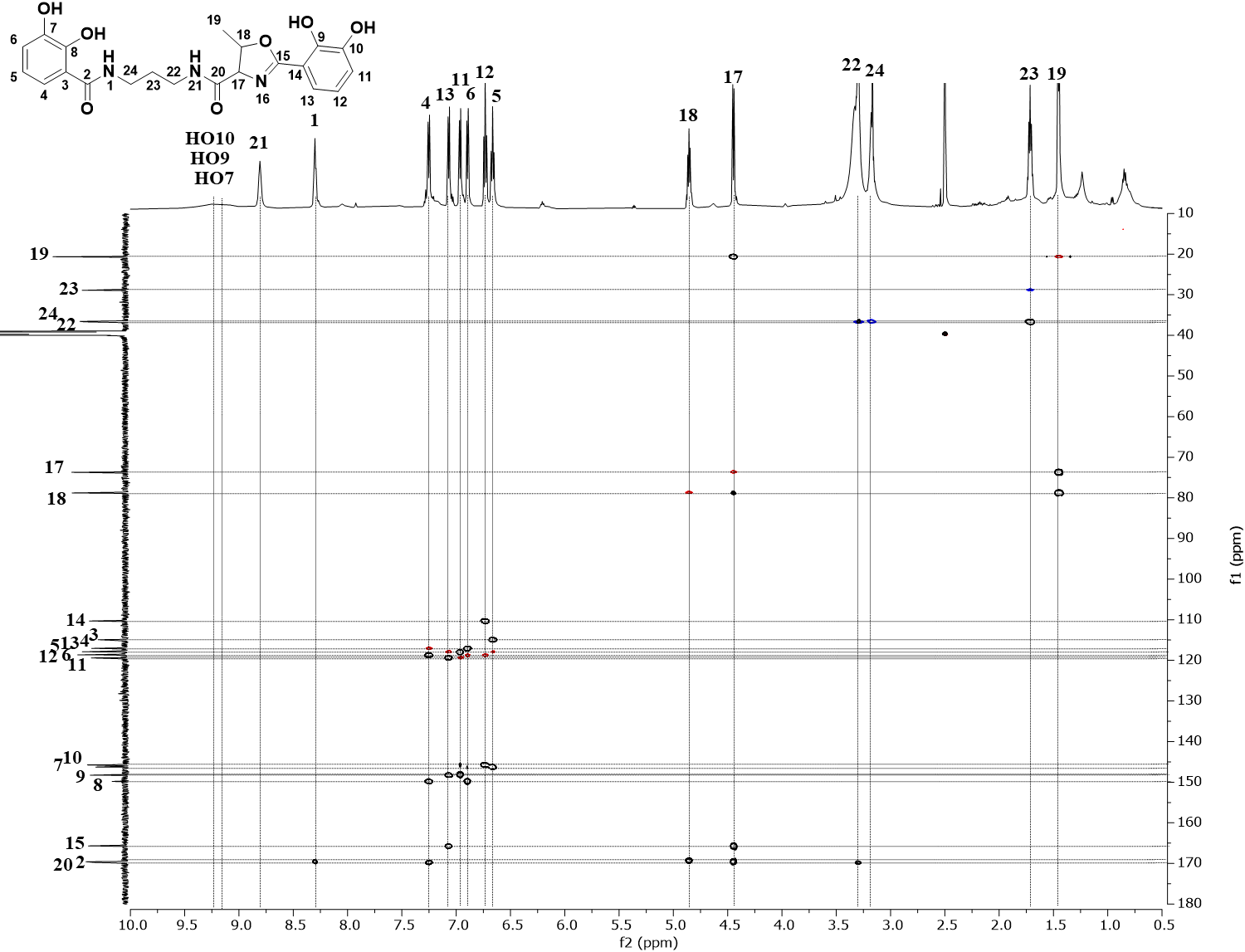


**S3.** HSQC + HMBC (600 MHz, DMSO-*d*_6_) spectrum of serratiochelin A (**1**)


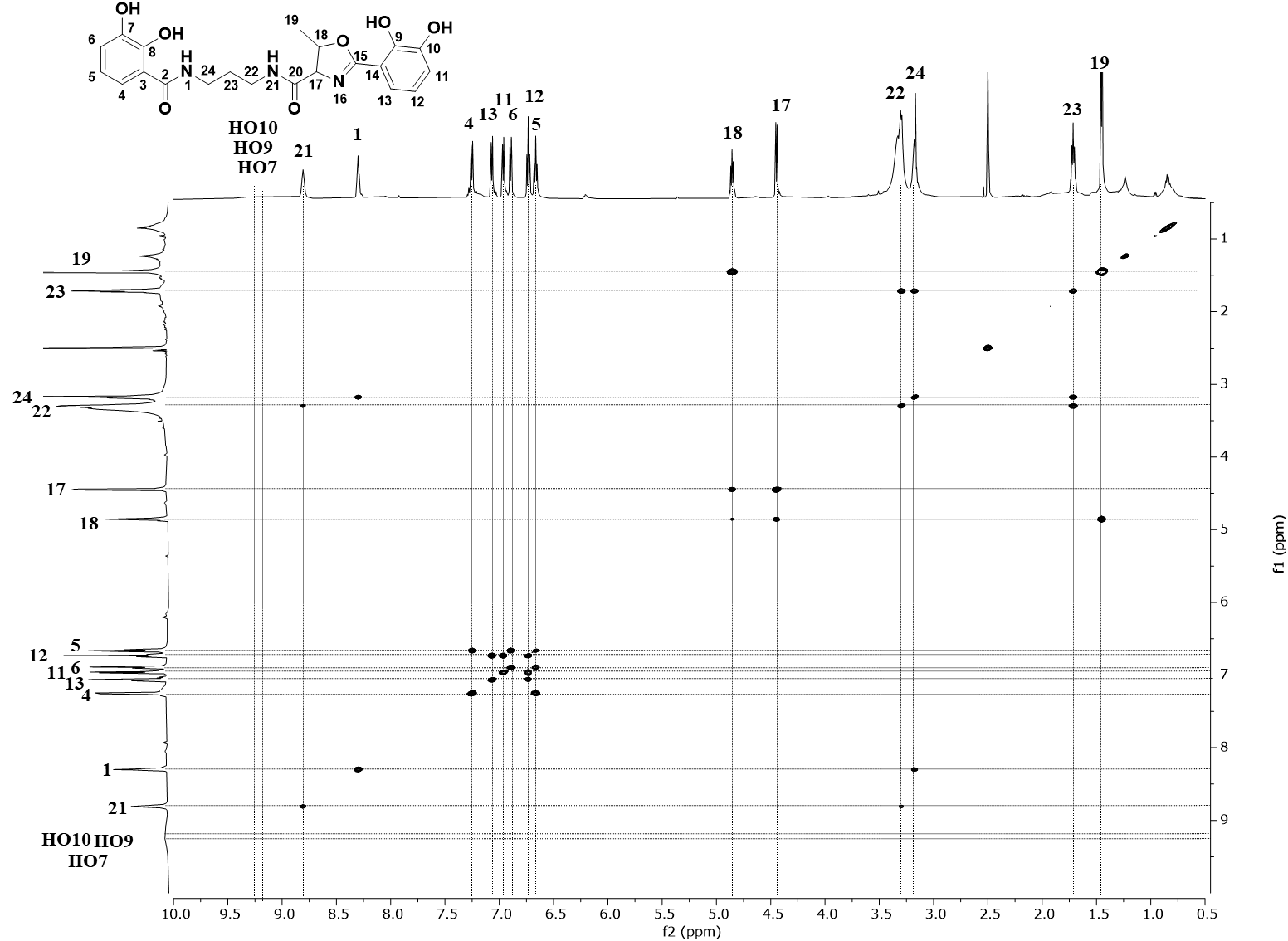


**S4.** COSY (600 MHz, DMSO-*d*_6_) spectrum of serratiochelin A (**1**)


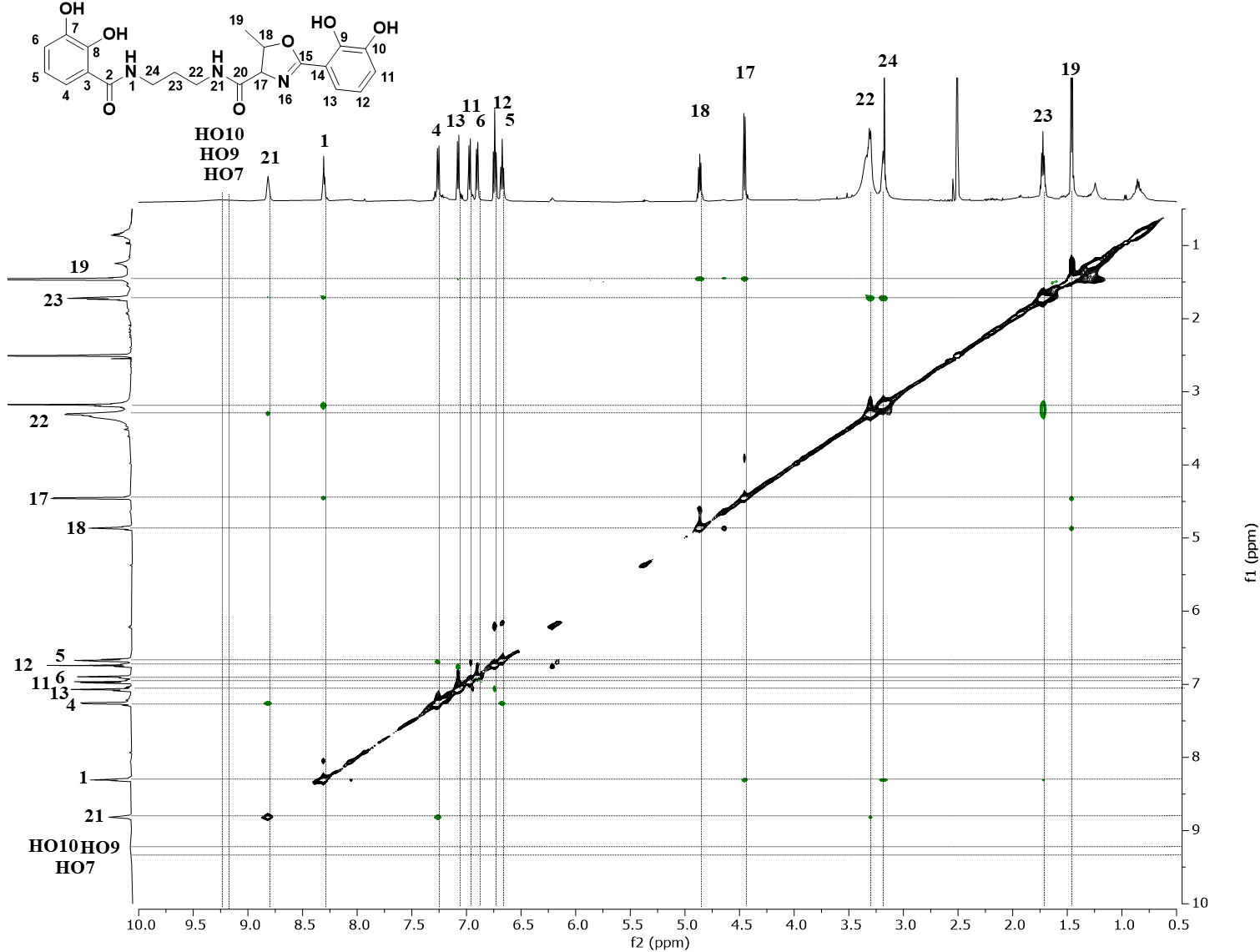


**S5.** ROESY (600 MHz, DMSO-*d*_6_) spectrum of serratiochelin A (**1**)


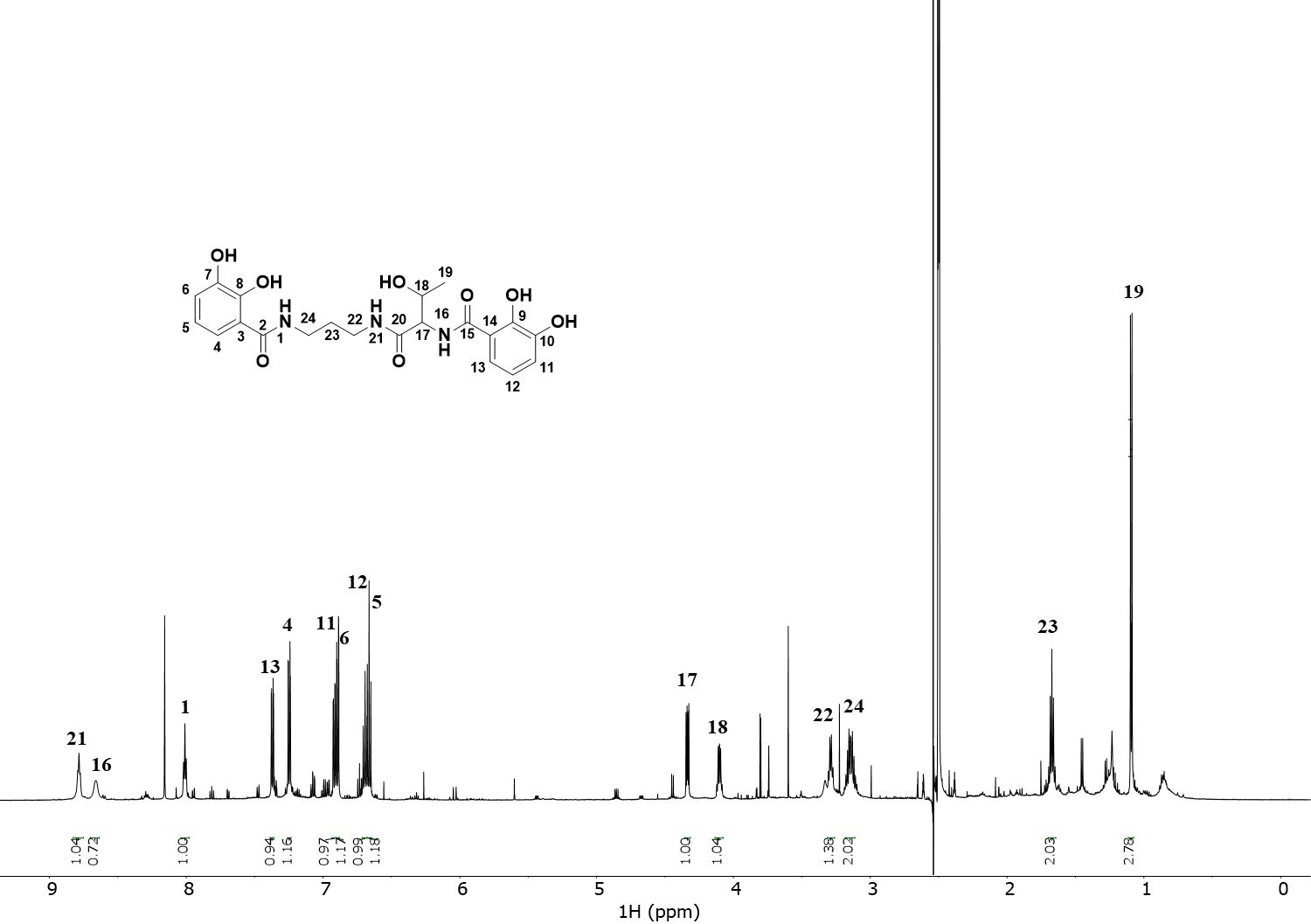


**S6.** ^1^H NMR (600 MHz, DMSO-*d*_6_) spectrum of serratiochelin C (**2**)


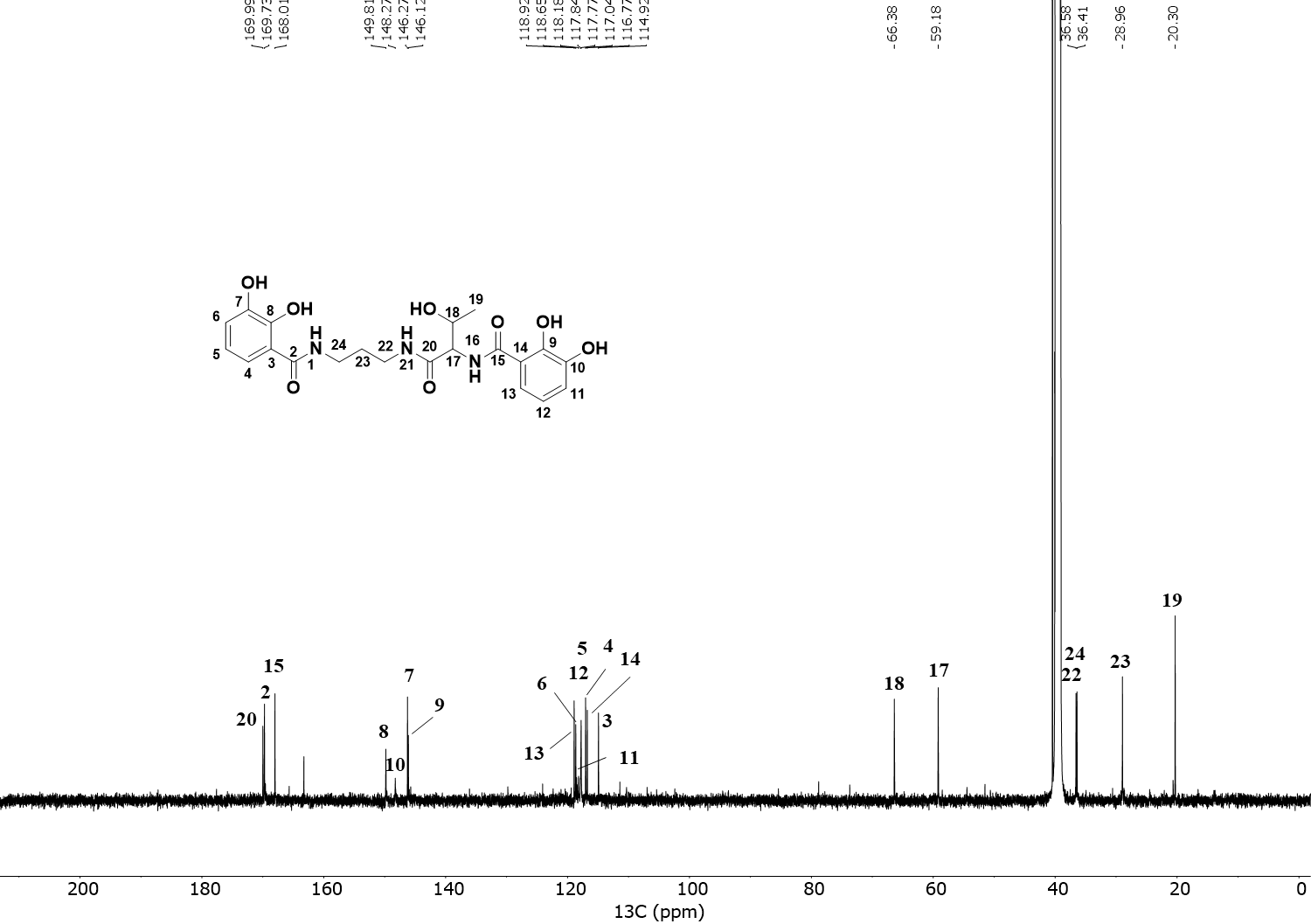


**S7.** ^13^C (151 MHz, DMSO-*d*_6_) spectrum of serratiochelin C (**2**)


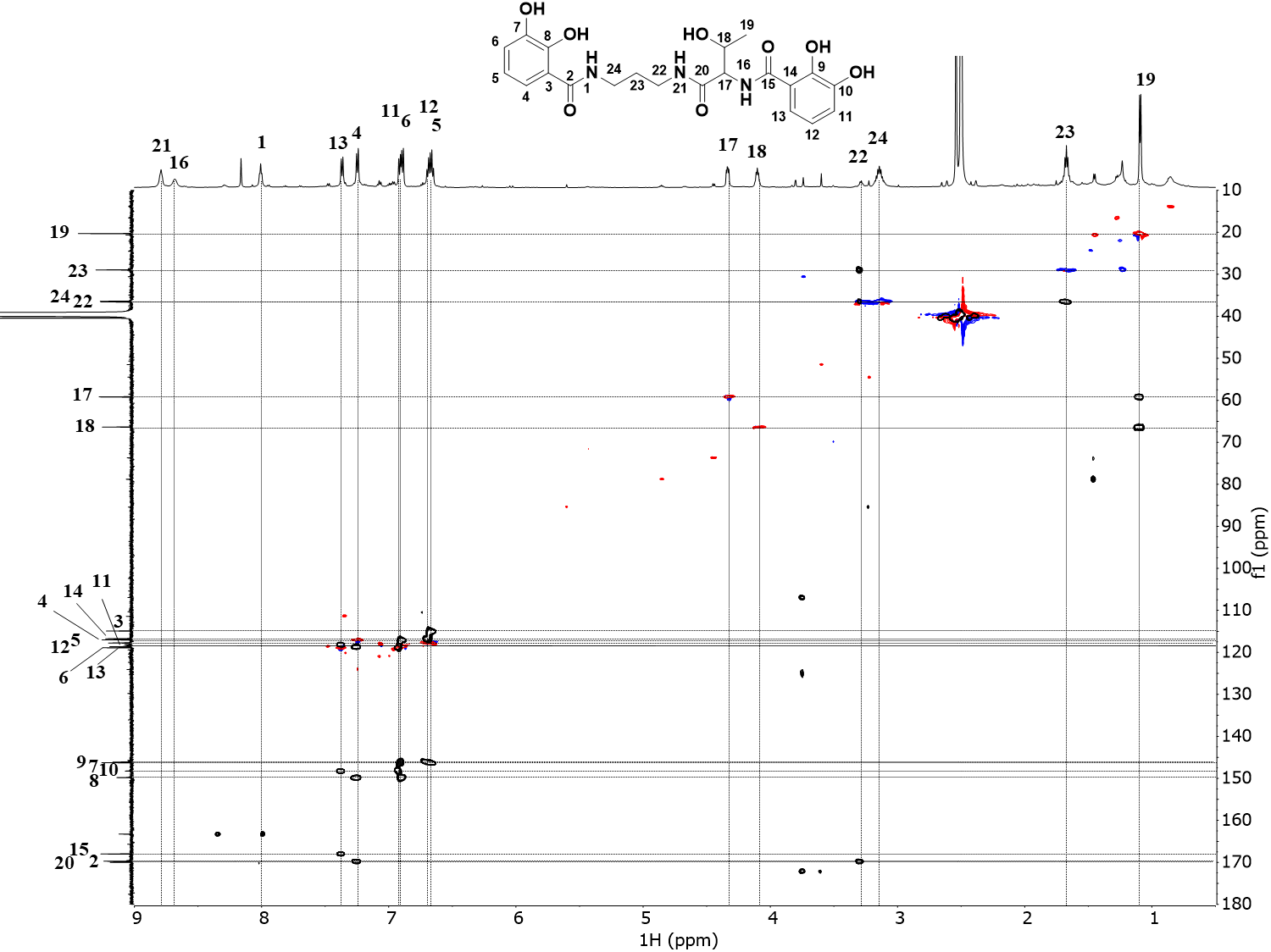


**S8.** HSQC + HMBC (600 MHz, DMSO-*d*_6_) spectrum of serratiochelin C (**2**)


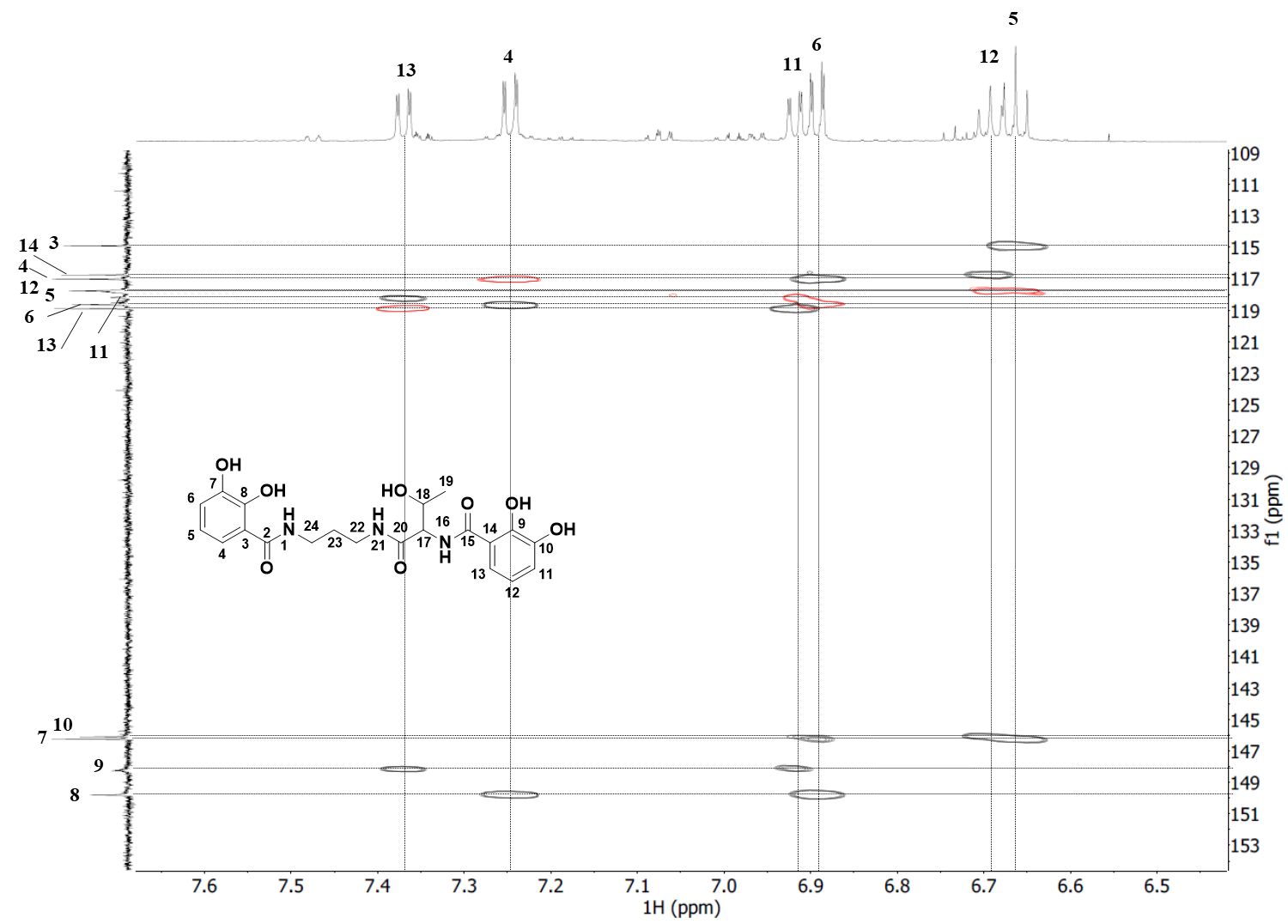


**S9.** HSQC + HMBC (600 MHz, DMSO-*d*_6_) spectrum of serratiochelin C (**2**), zoomed in crowded area


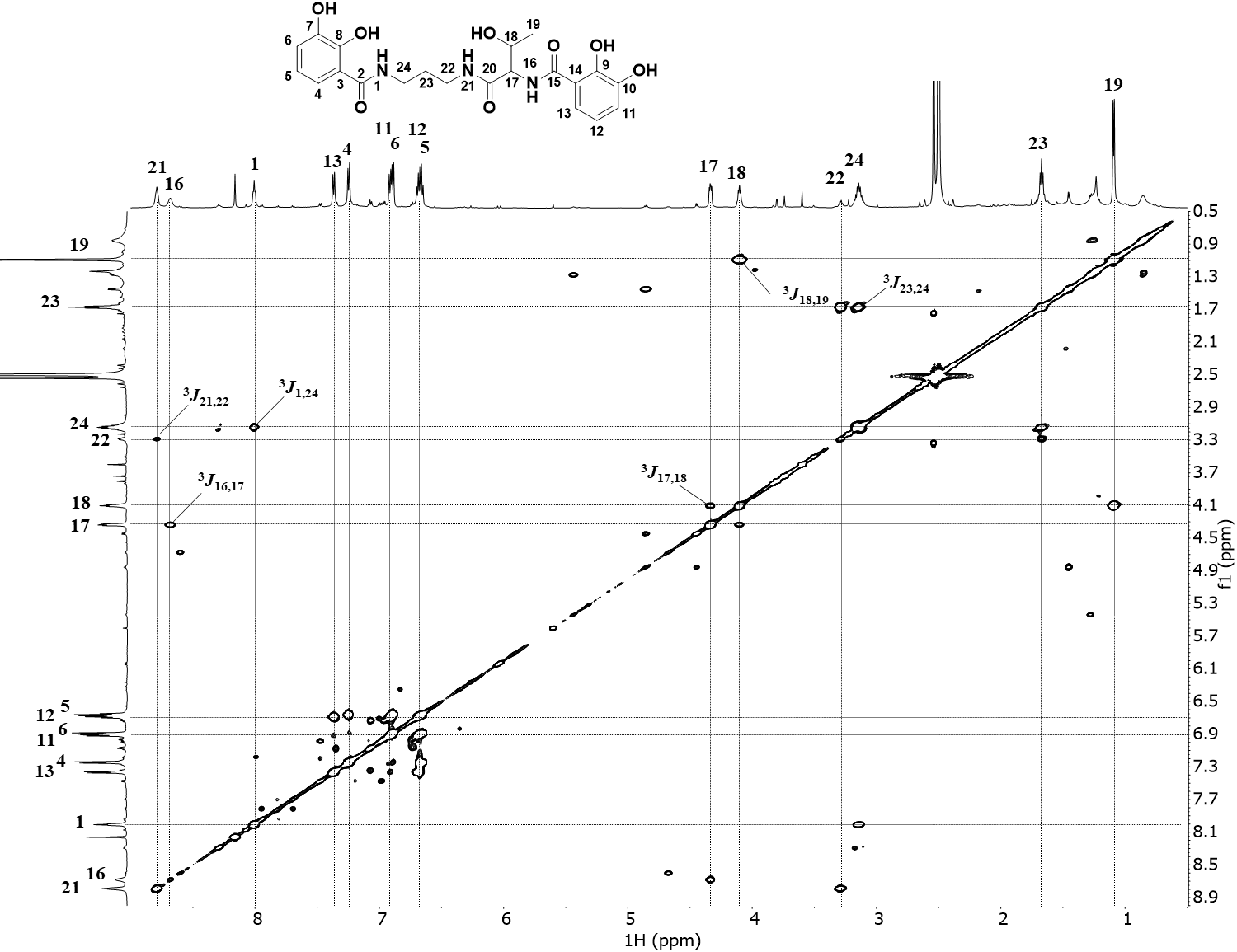


**S10.** COSY (600 MHz, DMSO-*d*_6_) spectrum of serratiochelin C (**2**)


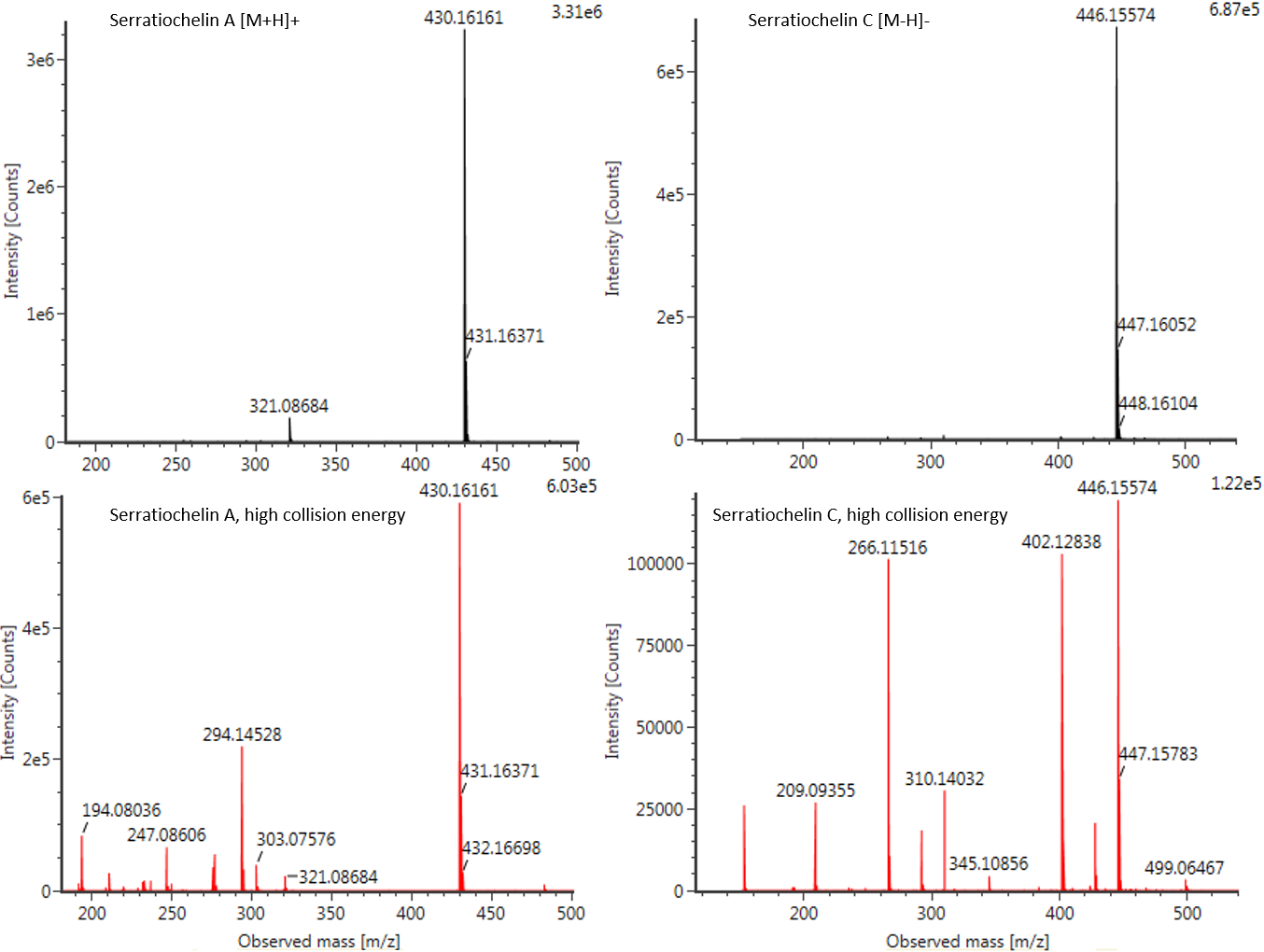


**S11**. Mass spectra of Serratiochelin A (**1**) (ESI+, left) and Serratiochelin C (**2**) (ESI-, right). The low-collision energy spectra are given in black and the high-collision energy spectra (20-80 eV ramp) in red below.


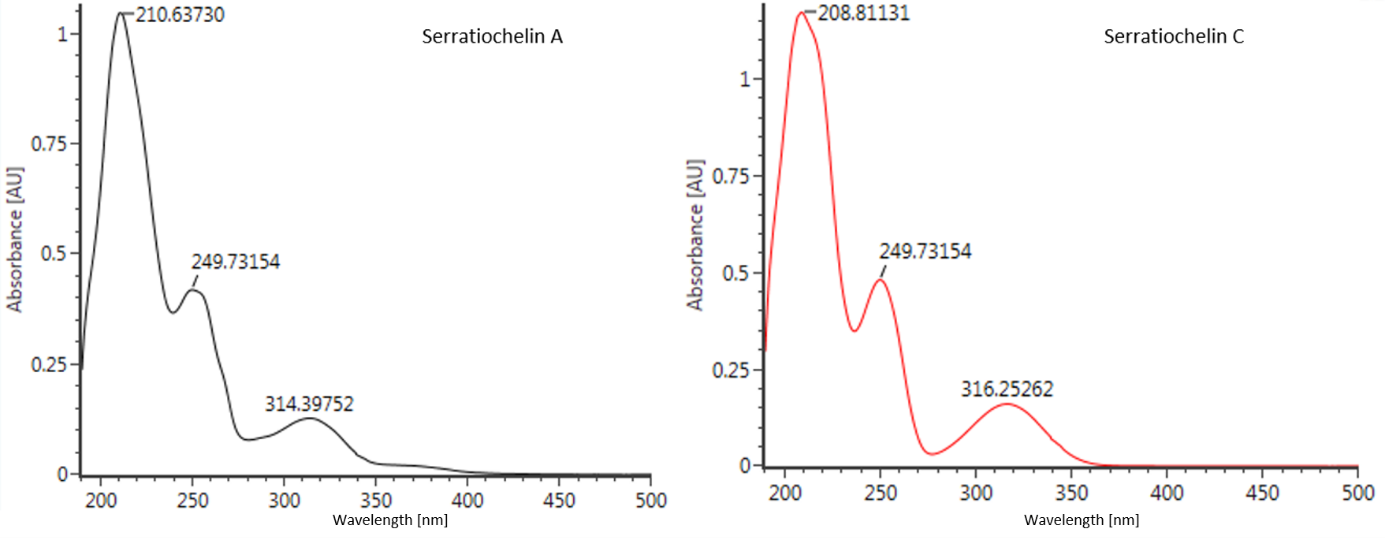


**S12**. UV/Vis spectra of Serratiochelin A (**1**) and C (**2**) in acetonitrile:water +1% (v/v) formic acid.


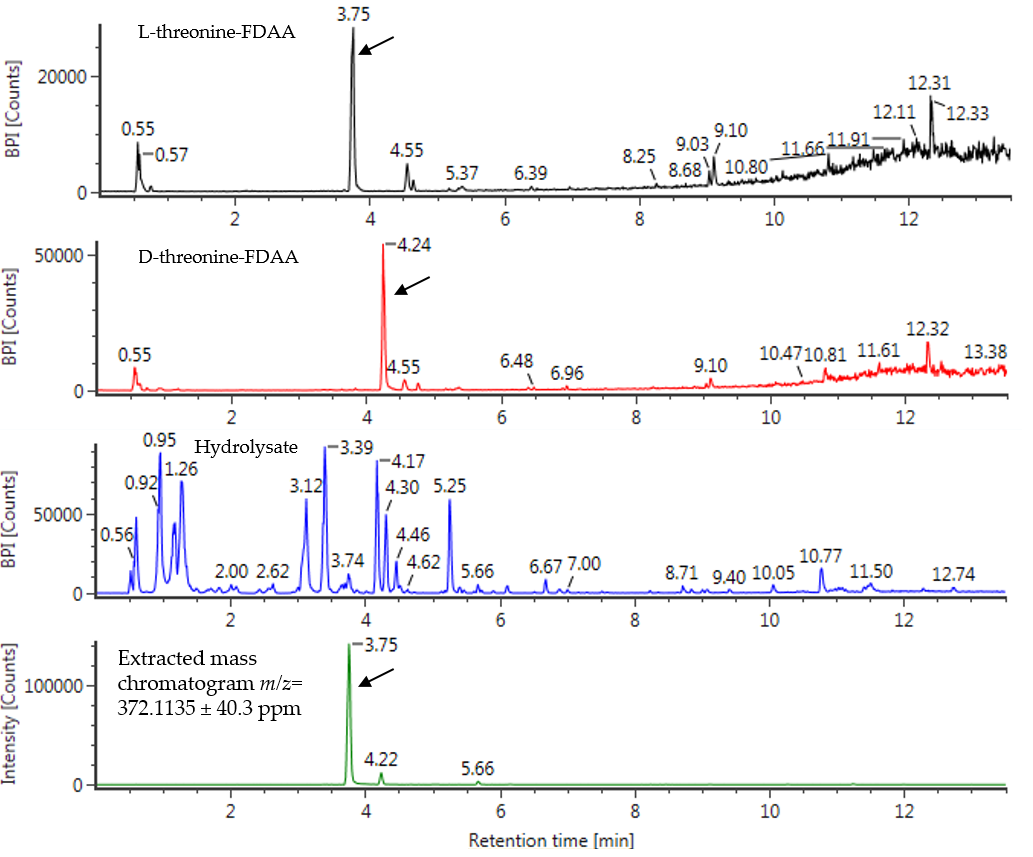


**S13**. Results of the threonine derivatisation using FDAA. The reactions were analysed using UHPLC-IMS-MS. At the top of the figure in black the chromatogram of L-threonine and below in red D-threonine is given. The chromatogram of the derivatised hydrolysate of serratiochelin A is given in blue (3^rd^ from the top) and at the bottom, the extracted mass-chromatogramm for FDAA-threonine adduct (C_13_H_17_N_5_O_8_, calculated monoisotopic mass: 371.1077 u) from the derivatised hydrolysate is given. Comparing the retention times of the L-and D-threonine references we conclude that L-threonine is the present configuration of threonine in serratiochelin A (**1**). The FDAA-threonine peaks are indicated by the black arrows.


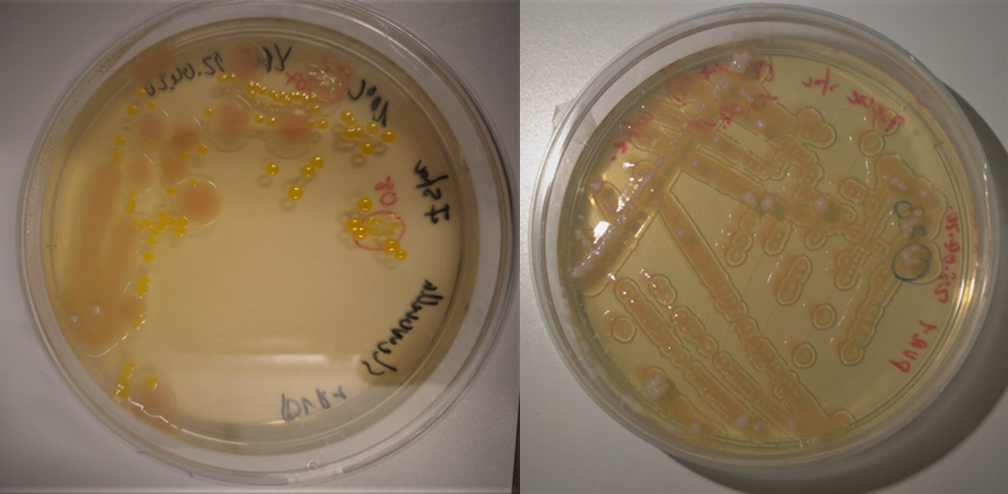


**S14**. Left: Non-axenic glycerol stock streaked on DVR1 agar displaying three morphologically different bacteria. Through 16S rRNA sequencing the colonies were shown to be *Leifsonia* sp. (yellow colonies), *Shewanella* (pink colonies) and *Serratia* sp. (white colonies). Right: Streak-out of liquid culture (3 days) started by the non-axenic glycerol stock showed *Serratia* sp. (white colonies) growing on top of the *Shewanella* sp. (light pink colonies).

**S15**. Consensus sequence of *Shewanella* sp.

Multiple sequences of forward and reverse reads were assembled, and the assembly was manually corrected where this was possible. The consensus sequence was used to conduct a Nucleotide BLAST with the nucleotide collection (nr/nt) database, excluding uncultured/environmental sample sequences was conducted giving exclusively hits for *Shewanella* sp. bacteria. The best match (date 20.05.20) was *Shewanella* sp. strain DZ-02-04-aga 16S ribosomal RNA gene, partial sequence (Accession number MK577329), with 100% identity.

>Shewanella_consensus TGCAGTCGAGCGGTAACACAAGGGAGCTTGCTCCTGAGGTGACGAGCGGCGGACGGGTGAGTAATGCCTAGGGATCTGCCCAGTCGAGGGGGATAACAGTTGGAAACGACTGCTAATACCGCATACGCCCTACGGGGGAAAGGAGGGGACCTTCGGGCCTTCCGCGATTGGATGAACCTAGGTGGGATTAGCTAGTTGGTGAGGTAATGGCTCACCAAGGCGACGATCCCTAGCTGTTCTGAGAGGATGATCAGCCACACTGGGACTGAGACACGGCCCAGACTCCTACGGGAGGCAGCAGTGGGGAATATTGCACAATGGGGGAAACCCTGATGCAGCCATGCCGCGTGTGTGAAGAAGGCCTTCGGGTTGTAAAGCACTTTCAGTAGGGAGGAAAGGTAGCGTGTTAATAGCACGTTACTGTGACGTTACCTACAGAAGAAGGACCGGCTAACTCCGTGCCAGCAGCCGCGGTAATACGGAGGGTCCGAGCGTTAATCGGAATTACTGGGCGTAAAGCGTGCGCAGGCGGTTTGTTAAGCCAGATGTGAAATCCCCGGGCTCAACCTGGGAATTGCATTTGGAACTGGCGAACTAGAGTCTTGTAGAGGGGGGTAGAATTCCAGGTGTAGCGGTGAAATGCGTAGATATCTGGAGGAATACCGGTGGCGAAGGCGGCCCCCTGGACAAAGACTGACGCTCATGCACGAAAGCGTGGGGAGCAAACAGGATTAGATACCCTGGTAGTCCACGCCGTAAACGATGTCTACTCGGAGTTTGGTGACTTAGTCACTGGGCTCCCAAGCTAACGCATTAAGTAGACCGCCTGGGGAGTACGGCCGCAAGGTTAAAACTCAAATGAATTGACGGGGGCCCGCACAAGCGGTGGAGCATGTGGTTTAATTCGATGCAACGCGAAGAACCTTACCTACTCTTGACATCCACAGAAGAGACCAGAGATGGACTTGTGCCTTCGGGAACTGTGAGACAGGTGCTGCATGGCTGTCGTCAGCTCGTGTTGTGAAATGTTGGGTTAAGTCCCGCAACGAGCGCAACCCCTATCCTTATTTGCCAGCACGTAATGGTGGGAACTCTAGGGAGACTGCCGGTGATAAACCGGAGGAAGGTGGGGACGACGTCAAGTCATCATGGCCCTTACGAGTAGGGCTACACACGTGCTACAATGGCGTATACAGAGGGTTGCAAAGCCGCGAGGTGGAGCTAATCTCACAAAGTACGTCGTAGTCCGGATCGGAGTCTGCAACTCGACTCCGTGAAGTCGGAATCGCTAGTAATCGTGGATCAGAATGCCACGGTGAATACGTTCCCGGGCCTTGTACACACCGCCCGTCACACCATGGGAGTGGGCTGCAAAAGAAGTGGGTAGTTTAACCTTCGGGAGAACGCTC

**S16**. Consensus sequence of *Serratia* sp.

Multiple sequences of forward and reverse reads were assembled, and the assembly was manually corrected where this was possible. The consensus sequence was used to conduct a Nucleotide BLAST with the nucleotide collection (nr/nt) database, excluding uncultured/environmental sample sequences was conducted giving exclusively hits for *Serratia* sp. bacteria. The best match (date 20.05.20) was *Serratia plymuthica* PRI-2C chromosome, complete genome (Accession number CP015613), with 100% identity.

>Serratia_consensus AAGCGCCCTCCCGAAGGTTAAGCTACCTACTTCTTTTGCAACCCACTCCCATGGTGTGACGGGCGGTGTGTACAAGGCCCGGGAACGTATTCACCGTAGCATTCTGATCTACGATTACTAGCGATTCCGACTTCATGGAGTCGAGTTGCAGACTCCAATCCGGACTACGACGTACTTTATGAGGTCCGCTTGCTCTCGCGAGTTCGCTTCTCTTTGTATACGCCATTGTAGCACGTGTGTAGCCCTACTCGTAAGGGCCATGATGACTTGACGTCATCCCCACCTTCCTCCGGTTTATCACCGGCAGTCTCCTTTGAGTTCCCGACCGAATCGCTGGCAACAAAGGATAAGGGTTGCGCTCGTTGCGGGACTTAACCCAACATTTCACAACACGAGCTGACGACAGCCATGCAGCACCTGTCTCAGAGTTCCCGAAGGCACTAAGCTATCTCTAGCGAATTCTCTGGATGTCAAGAGTAGGTAAGGTTCTTCGCGTTGCATCGAATTAAACCACATGCTCCACCGCTTGTGCGGGCCCCCGTCAATTCATTTGAGTTTTAACCTTGCGGCCGTACTCCCCAGGCGGTCGATTTAACGCGTTAGCTCCGGAAGCCACGCCTCAAGGGCACAACCTCCAAATCGACATCGTTTACAGCGTGGACTACCAGGGTATCTAATCCTGTTTGCTCCCCACGCTTTCGCACCTGAGCGTCAGTCTTTGTCCAGGGGGCCGCCTTCGCCACCGGTATTCCTCCAGATCTCTACGCATTTCACCGCTACACCTGGAATTCTACCCCCCTCTACAAGACTCTAGCTTGCCAGTTTCAAATGCAGTTCCCACGTTAAGCGCGGGGATTTCACATCTGACTTAACAAACCGCCTGCGTGCGCTTTACGCCCAGTAATTCCGATTAACGCTTGCACCCTCCGTATTACCGCGGCTGCTGGCACGGAGTTAGCCGGTGCTTCTTCTGCGAGTAACGTCAATGCAATGTGCTATTAACACATTACCCTTCCTCCTCGCTGAAAGTGCTTTACAACCCTAAGGCCTTCTTCACACACGCGGCATGGCTGCATCAGGCTTGCGCCCATTGTGCAATATTCCCCACTGCTGCCTCCCGTAGGAGTCTGGACCGTGTCTCAGTTCCAGTGTGGCTGGTCATCCTCTCAGACCAGCTAGGGATCGTCGCCTAGGTGAGCCATTACCCCACCTACTAGCTAATCCCATCTGGGCACATCTGATGGCGTGAGGCCCGAAGGTCCCCCACTTTGGTCCGTAGACGTTATGCGGTATTAGCTACCGTTTCCAGTAGTTATCCCCCTCCATCAGGCAGTTTCCCAGACATTACTCACCCGTCCGCCGCTCGTCACCCAGAGAGCAAGCTCTCCTGTGCTACCGCTCGACTTGCAT

**S17**. Consensus sequence of *Leifsonia* sp.

Multiple sequences of forward and reverse reads were assembled, and the assembly was manually corrected where this was possible. The consensus sequence was used to conduct a Nucleotide BLAST with the nucleotide collection (nr/nt) database, excluding uncultured/environmental sample sequences was conducted giving hits for bacteria of different genera, mainly *Salinibacterium* sp., *Leifsonia* sp., *Agreia* sp., and other un-identified bacteria of marine origin and Actinobacteria, all with % identity above 99%. The best hit (date 20.05.20) was surprisingly found to be *Pseudomonas* sp. AW15 16S ribosomal RNA gene, partial sequence (Accession number FJ362501, 99.93% identity), but the identity of this sequence is questionable as it has no hits for other *Pseudomonas* sp. through BLAST. The second best hit for our sequence was for *Salinibacterium* sp. strain DZ-02-03-aga 16S ribosomal RNA gene, partial sequence (Accession number MK577334, 99.86% identity).

>Leifsonia_consensus TGCAGTCGAACGATGAAGCTGGAGCTTGCTCTGGTGGATTAGTGGCGAACGGGTGAGTAACACGTGAGTAACCTGCCCTTGACTCTGGAATAAGCGTTGGAAACGACGTCTAATACCGGATACGAGCTTCCGCCGCATGGTGAGGAGCTGGAAAGAATTTCGGTCAAGGATGGACTCGCGGCCTATCAGGTAGTTGGTGAGGTAATGGCTCACCAAGCCTACGACGGGTAGCCGGCCTGAGAGGGTGACCGGCCACACTGGAACTGAGACACGGTCCAGACTCCTACGGGAGGCAGCAGTGGGGAATATTGCACAATGGGCGCAAGCCTGATGCAGCAACGCCGCGTGAGGGACGACGGCCTTCGGGTTGTAAACCTCTTTTAGTAGGGAAGAAGCGAAAGTGACGGTACCTGCAGAAAAAGCACCGGCTAACTACGTGCCAGCAGCCGCGGTAATACGTAGGGTGCAAGCGTTATCCGGAATTATTGGGCGTAAAGAGCTCGTAGGCGGTTTGTCGCGTCTGCTGTGAAAACTGGGGGCTCAACCCCCAGCCTGCAGTGGGTACGGGCAGACTAGAGTGCGGTAGGGGAGATTGGAATTCCTGGTGTAGCGGTGGAATGCGCAGATATCAGGAGGAACACCAATGGCGAAGGCAGATCTCTGGGCCGTTACTGACGCTGAGGAGCGAAAGCATGGGGAGCGAACAGGATTAGATACCCTGGTAGTCCATGCCGTAAACGTTGGGAACTAGATGTAGGGGCCATTCCACGGTTTCTGTGTCGCAGCTAACGCATTAAGTTCCCCGCCTGGGGAGTACGGCCGCAAGGCTAAAACTCAAAGGAATTGACGGGGGCCCGCACAAGCGGCGGAGCATGCGGATTAATTCGATGCAACGCGAAGAACCTTACCAAGACTTGACATATACGAGAACGGGCTAGAAATAGTTCACTCTTTGGACACTCGTAAACAGGTGGTGCATGGTTGTCGTCAGCTCGTGTCGTGAGATGTTGGGTTAAGTCCCGCAACGAGCGCAACCCTCGTTCTTTGTTGCCAGCACGTAATGGTGGGAACTCAAAGGAGACTGCCGGGGTCAACTCGGAGGAAGGTGGGGATGACGTCAAATCATCATGCCCCTTATGTCTTGGGCTTCACGCATGCTACAATGGCCGATACAAAGGGCTGCAATACCGCGAGGTAGAGCGAATCCCAAAAAGTCGGTCTCAGTTCGGATTGAGGTCTGCAACTCGACCTCATGAAGTCGGAGTCGCTAGTAATCGCAGATCAGCAACGCTGCGGTGAATACGTTCCCGGGCCTTGTACACACCGCCCGTCAAGTCATGAAAGTCGGTAACACCCGAAGCCAGTGGCCTAACCCGCAAG
